# ﻿Notes on twelve species of jumping spiders from Hainan Island, China (Araneae, Salticidae)

**DOI:** 10.3897/zookeys.1167.105424

**Published:** 2023-06-15

**Authors:** Cheng Wang, Shuqiang Li

**Affiliations:** 1 Guizhou Provincial Key Laboratory for Biodiversity Conservation and Utilization in the Fanjing Mountain Region, Tongren University, Tongren, 554300 Guizhou, China Tongren University Tongren China; 2 Ministry of Education Key Laboratory for Ecology of Tropical Islands, College of Life Sciences, Hainan Normal University, 571158 Haikou, China Hainan Normal University Haikou China; 3 Institute of Zoology, Chinese Academy of Sciences, Beijing 100101, China Institute of Zoology, Chinese Academy of Sciences Beijing China

**Keywords:** Morphology, new taxa, salticid, southern China, taxonomy

## Abstract

Three new genera and eleven new species are reported from Hainan Island, China. The new genera are *Logunattus***gen. nov.**, including *L.dufui***sp. nov.** (♂), and the generotype *L.libaii***sp. nov.** (♂♀), *Qiongattusyuanyeae***gen. et sp. nov.** (♂♀), and *Spiralembolus***gen. nov.**, including the generotype *S.yinggeling***sp. nov.** (♂♀), and *S.yui***sp. nov.** (♂♀). Another six new species are *Carrhotusqingzhaoae***sp. nov.** (♂♀), *Gedealiangweii***sp. nov.** (♂♀), *Heliophanoidesmoi***sp. nov.** (♂), *Indopadillasongi***sp. nov.** (♂♀), *Myrmarachnemixiaoqii***sp. nov.** (♂♀), and *Nandiciusshihaitaoi***sp. nov.** (♂♀). The unknown female of the endemic species, *Pancoriushainanensis* Song & Chai, 1991 is also described for the first time. Diagnostic photos of these species are provided.

## ﻿Introduction

Hainan, the second-largest Chinese Island, has presented very high species diversity (Tang and Li 2010). As in most Chinese regions, the taxonomic study of jumping spiders from this island began at the end of the 20^th^ century (Song et al. 1988), but knowledge has rapidly increased in the last more than three decades, with the series of taxonomic studies and biodiversity surveys conducted (Song et al. 1988; Song 1991; Song and Chai 1991; Peng and Kim 1997; Song and Zhu 1998; Zhang and Li 2005; Peng and Li 2006; Guo 2011; Guo et al. 2011a, b; Barrion et al. 2013; Zhou and Li 2013; Xu et al. 2021; Wang and Li 2022b; Yu et al. 2022). To date, at least 123 salticid species including 47 endemics have been recorded from Hainan (Metzner 2023; Wang and Li 2022b; WSC 2023). However, there is no doubt that the true diversity of jumping spiders from this island remains insufficiently known, and numerous new species or newly recorded species will continue being discovered with further taxonomic studies and broad surveys. Moreover, like the worldwide current situation, the taxonomic study of jumping spiders from this island is also plagued by high rates of taxa known from a single-sex and some poorly known species lacking diagnostic drawings (Wang and Li 2022b).

The present work is the result of an ongoing taxonomic study of Hainan’s jumping spider specimens deposited at the Institute of Zoology, Chinese Academy of Sciences in Beijing (IZCAS), China, and Tongren University (TRU) in Tongren, China. Eleven species belonging to nine genera (including three new genera) are recognized as new to science. The unknown female of *Pancoriushainanensis* Song & Chai, 1991 was also found and is described herein for the first time.

## ﻿Materials and methods

Specimens were collected by beating shrubs, sieving, or hand collecting in the tropical rainforest of Hainan Island, China. They were preserved in 75% ethanol for morphological study. Specimens are deposited at the Institute of Zoology, Chinese Academy of Sciences in Beijing (**IZCAS**), China, and Tongren University (**TRU**) in Tongren, China. Methods follow those of Wang and Li (2021). All measurements are given in millimetres. Leg measurements are given as total length (femur, patella, tibia, metatarsus, tarsus). References to figures in the cited papers are listed in lowercase type (fig. or figs), and figures in this paper are noted with an initial capital (Fig. or Figs).

Abbreviations used in the text and figures are as follows: **ALE** anterior lateral eye; **AME** anterior median eye; **AERW** anterior eye row width; **AG** accessory gland; **AR** atrial ridge; **AS** anterior chamber of spermatheca; **At** atrium; **BG** Bennett’s gland; **BP** basal epigynal plate; **CA** cymbial apophysis; **CD** copulatory duct; **CO** copulatory opening; **DTA** dorsal tibial apophysis; **E** embolus; **EFL** eye field length; **FD** fertilization duct; **H** epigynal hood; **LP** lamellar process; **MS** median septum; **PERW** posterior eye row width; **PL** posterior lobe; **PLE** posterior lateral eye; **PS** posterior chamber of spermatheca; **bRTA** baso-retrolateral tibial apophysis; **dRTA** dorso-retrolateral tibial apophysis; **RTA** retrolateral tibial apophysis; **S** spermatheca; **SD** sperm duct; **SH** spermathecal head; **TB** tegular bump.

Institutional abbreviations: **IZCAS** Institute of Zoology, Chinese Academy of Sciences; **TRU** Tongren University.

## ﻿Taxonomy

**Family Salticidae Blackwall, 1841**

### ﻿Genus *Carrhotus* Thorell, 1891

#### ﻿ Carrhotus qingzhaoae sp. nov.

Taxon classificationAnimaliaAraneaeSalticidae

9D238101-F8F4-5072-B6F3-A4B28C4224F3

https://zoobank.org/36FC84A1-6B38-459B-A3CA-3DD0FF9D34BE

Figs 1 , 2

##### Type material.

***Holotype*** ♂ (TRU-JS 0682), China: Hainan: Ledong County, Jianfengling National Nature Reserve, Peak Mountain (18°43.11′N, 108°52.32′E, ca. 1400 m), 17.iv.2019, C. Wang & Y.F. Yang leg. ***Paratypes*** 1♂2♀ (TRU-JS 0683–0685), same data as holotype.

##### Etymology.

The specific name is after Mrs. Qingzhao Li (1084–1155), the most outstanding female writer in the history of Chinese literature; noun (name) in genitive case.

##### Diagnosis.

**Figure 1.**
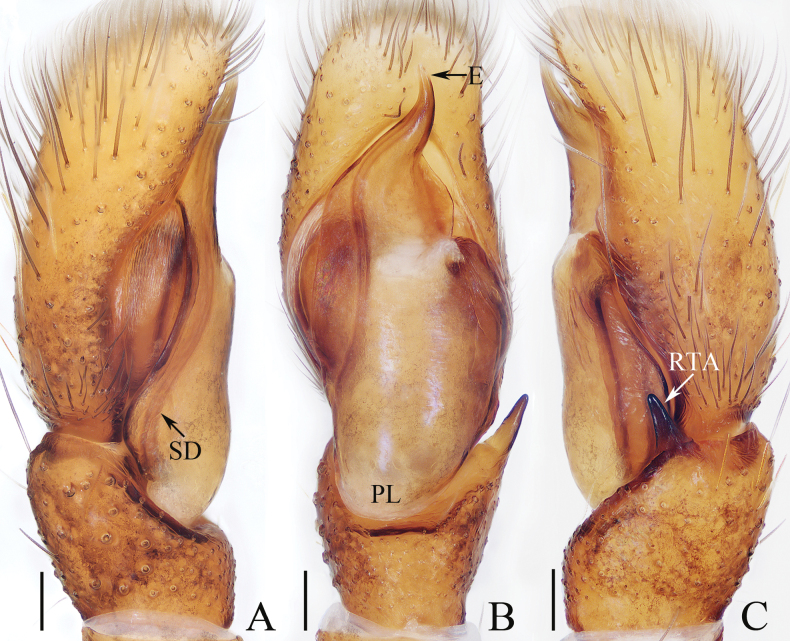
Male palp of *Carrhotusqingzhaoae* sp. nov., holotype **A** prolateral **B** ventral **C** retrolateral. Scale bars: 0.1 mm.

**Figure 2.**
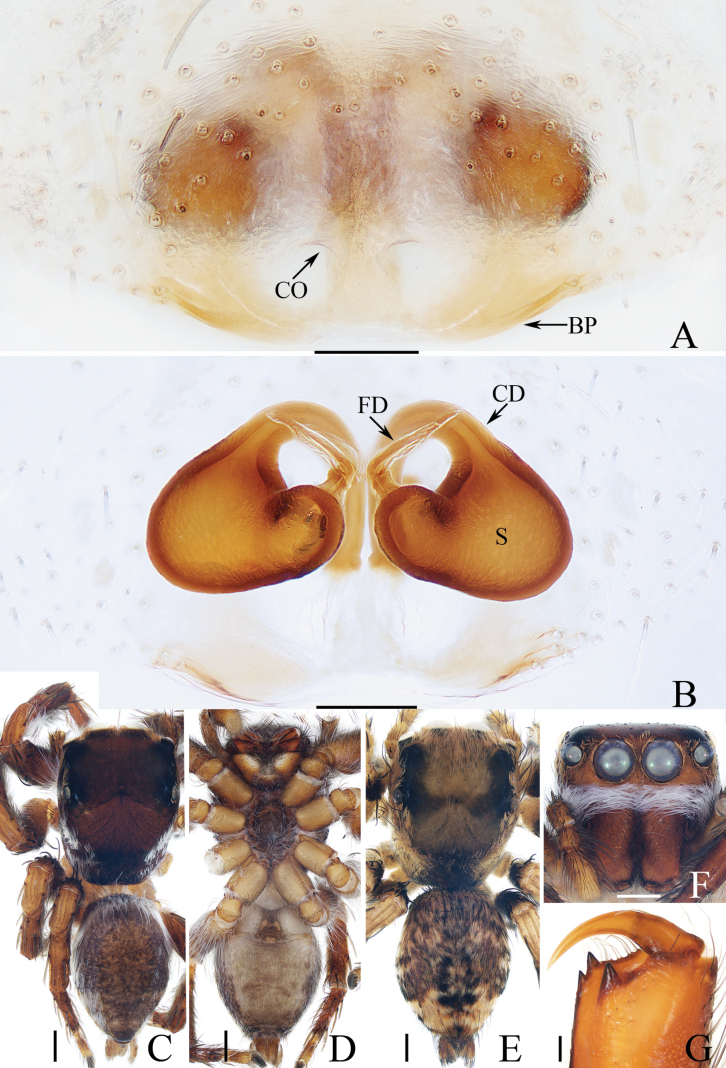
*Carrhotusqingzhaoae* sp. nov., male holotype and female paratype **A** epigyne, ventral **B** vulva, dorsal **C** holotype habitus, dorsal **D** ditto, ventral **E** female paratype habitus, dorsal **F** holotype carapace, frontal **G** holotype chelicera, posterior. Scale bars: 0.1 mm (**A, B, G**); 0.5 mm (**C–F**).

The male of *Carrhotusqingzhaoae* sp. nov. resembles that of *C.atratus* Satkunanathan & Benjamin, 2022 in the general shape of palp, but differs in: (1) the embolus being narrower than 1/5 the bulb width in ventral view (Fig. 1B), vs. ~ 1/3 the bulb width in *C.atratus* (Satkunanathan and Benjamin 2022: fig. 7C); (2) the RTA is straight and tapered in retrolateral view (Fig. 1C), vs. slightly curved and acutely narrowed distally in *C.atratus* (Satkunanathan and Benjamin 2022: figs 6F, 7D). The female resembles that of *C.samchiensis* Jastrzębski, 1999 in having very similar epigyne, but it can be easily distinguished by the following: (1) the copulatory openings are located posteriorly (Fig. 2A), vs. located anteromedially in *C.samchiensis* (Jastrzębski 1999: fig. 20); (2) the copulatory ducts are greater than spermathecal width in length (Fig. 2B), vs. ca. the equal in length to spermathecal width in *C.samchiensis* (Jastrzębski 1999: fig. 21).

##### Description.

**Male** (Figs 1, 2C, D, F, G). Total length 5.03. Carapace 2.46 long, 2.03 wide. Abdomen 2.43 long, 1.60 wide. Clypeus 0.12 high. Eye sizes and inter-distances: AME 0.53, ALE 0.30, PLE 0.28, AERW 1.83, PERW 1.93, EFL 1.07. Legs: I 5.31 (1.55, 0.90, 1.25, 0.93, 0.68), II 4.83 (1.50, 0.85, 1.10, 0.83, 0.55), III 5.39 (1.65, 0.88, 1.23, 1.00, 0.63), IV 5.47 (1.60, 0.88, 1.28, 1.13, 0.58). Carapace red-brown to dark brown, covered with dense white setae on the later sides of thorax, and clypeus; fovea dark, linear, longitudinal. Chelicerae red-brown, each with two promarginal teeth and one retromarginal tooth. Endites pale to yellow, setose, widened distally. Labium red-brown to dark brown, with pale anterior margin. Sternum red-brown, covered with long white setae. Legs dark yellow to brown, setose and spinous. Abdomen almost oval, dorsum violet-brown, covered with dense, anterior, and lateral long setae, and covered by elliptical scutum extending from anterior 1/5 to the terminus and ca. half the abdomen width; venter grey to brown, setose. Palp (Fig. 1A–C). Tibia slightly longer than wide, with straight, tapered RTA directed towards ~ 11:00 o’clock position apically in retrolateral view; cymbium ~ 2× longer than wide in ventral view, with thin setae of various lengths; bulb elongated, with well-developed posterior lobe extending postero-prolaterally; embolus short, tapered, originates from the apical portion of bulb, curved towards prolateral side medially, with rather pointed tip.

**Female** (Fig. 2A, B, E). Total length 5.49. Carapace 2.78 long, 2.41 wide. Abdomen 2.74 long, 2.07 wide. Clypeus 0.13 high. Eye sizes and inter-distances: AME 0.55, ALE 0.33, PLE 0.30, AERW 2.15, PERW 2.33, EFL 1.33. Legs: I 5.10 (1.60, 1.00, 1.20, 0.70, 0.60), II 4.81 (1.58, 0.88, 1.00, 0.75, 0.60), III 5.71 (1.80, 1.00, 1.23, 1.05, 0.63), IV 5.87 (1.83, 0.98, 1.25, 1.18, 0.63). Carapace similar to that of male, except paler, covered with dense yellow setae and sparse dark, long setae, without white setae on the lateral of thorax, and clypeus. Abdomen oval, dorsum yellow to brown, setose, with yellow, dark markings of setae. Epigyne (Fig. 2A, B). Wider than long, with arc-shaped basal plate almost as wide as epigyne; copulatory openings posteriorly located, separated from each other less than their width; copulatory ducts thin, paralleled extending anteromedially and followed by ~ 110° curves, and connected to the anterior portions of spermathecae distally; spermathecae irregular, separated from each other less than 1/6 their width.

##### Distribution.

Known only from the type locality in Hainan Island, China.

### ﻿Genus *Gedea* Simon, 1902

#### ﻿ Gedea liangweii sp. nov.

Taxon classificationAnimaliaAraneaeSalticidae

9C40DF6C-BA3A-5B87-8BF5-0C1B907DDA23

https://zoobank.org/B66459A9-0D5F-4F4F-A1DF-9020E28E99DD

Figs 3 , 4

##### Type material.

***Holotype*** ♂ (IZCAS-Ar44497), China: Hainan: Ledong County, Jianfengling National Nature Reserve (18°44.73′N, 108°49.63′E, ca. 1240 m), 17.viii.2010, G. Tang leg. ***Paratypes*** 1♂1♀ (IZCAS-Ar44498–44499), same data as holotype; 1♂ (IZCAS-Ar44500), Jianfeng Botanical Garden (18°44.27′N, 108°51.50′E, ca. 910 m), 28.iv.2009, G. Tang leg.; 1♂ (IZCAS-Ar44501), Wuzhishan City, Wuzhishan National Nature Reserve, 7.viii.2007, C.X. Wang leg.

##### Etymology.

The specific name is after Prof. Liang Wei from Hainan Normal University, China; noun (name) in genitive case.

##### Diagnosis.

**Figure 3.**
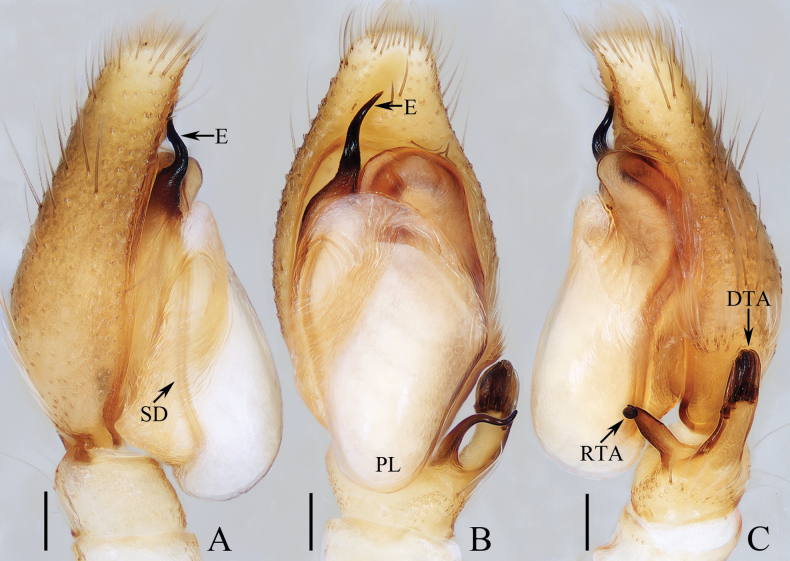
Male palp of *Gedealiangweii* sp. nov., holotype **A** prolateral **B** ventral **C** retrolateral. Scale bars: 0.1 mm.

**Figure 4.**
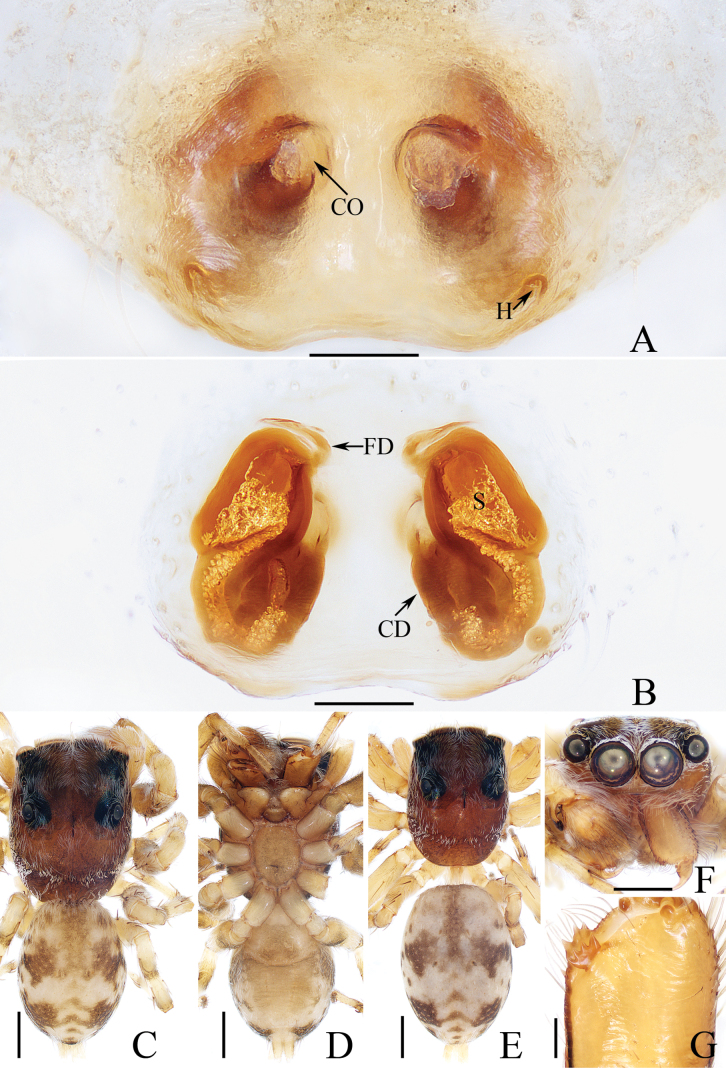
*Gedealiangweii* sp. nov., male holotype and female paratype **A** epigyne, ventral **B** vulva, dorsal **C** holotype habitus, dorsal **D** ditto, ventral **E** female paratype habitus, dorsal **F** holotype carapace, frontal **G** holotype chelicera, posterior. Scale bars: 0.1 mm (**A, B, G**); 0.5 mm (**C–F**).

*Gedealiangweii* sp. nov. closely resembles that of *G.daoxianensis* Song & Gong, 1992 in having very similar habitus and copulatory organs, but it can be easily distinguished by the following: (1) the DTA bears six digitiform apophyses distally in retrolateral view (Fig. 3C), vs. several long setae-like apophyses in *G.daoxianensis* (Song and Gong 1992: fig. 6); (2) the copulatory openings are anteriorly located (Fig. 4A), vs. posteriorly located in *G.daoxianensis* (Song and Gong 1992: fig. 3); (3) the copulatory ducts are curved ~ 180° medially (Fig. 4B), vs. curved ~ 100° in *G.daoxianensis* (Song and Gong 1992: fig. 4).

##### Description.

**Male** (Figs 3, 4C, D, F, G). Total length 3.26. Carapace 1.72 long, 1.35 wide. Abdomen 1.57 long, 1.20 wide. Clypeus 0.07 high. Eye sizes and inter-distances: AME 0.37, ALE 0.26, PLE 0.21, AERW 1.24, PERW 1.11, EFL 0.80. Legs: I 3.48 (0.95, 0.65, 0.85, 0.63, 0.40), II 2.91 (0.90, 0.50, 0.63, 0.50, 0.38), III 3.33 (1.10, 0.50, 0.63, 0.70, 0.40), IV 3.23 (1.00, 0.43, 0.65, 0.75, 0.40). Carapace red-brown to dark brown, covered with dense yellow and white setae, with pair of indistinct, dark patches medially on eye field; fovea linear, longitudinal, dark. Chelicerae yellow, each with four promarginal teeth and one retromarginal fissidental tooth with four cusps. Endites paler than chelicerae, with sub-triangular distal processes extending laterally. Labium almost linguiform, bearing several dark setae at anterior margin. Sternum slightly longer than wide, with straight anterior margin, covered with paler thin setae. Legs yellow to brown, spinous. Abdomen elongate-oval, dorsum with two pairs of muscle depressions anteromedially and alternate dark brown and pale yellow transverse bands; venter pale-yellow to grey. Palp (Fig. 3A–C). Tibia wider than long, with curved, tapered RTA coiled into a circle distally in retrolateral view, and platelike DTA bearing six digitiform apophyses at distal end; cymbium almost two times longer than wide; bulb swollen, with well-developed posterior lobe extending downward; embolus strongly sclerotized, tapered, and curved, originates from antero-prolateral portion of bulb, with rather pointed tip.

**Female** (Fig. 4A, B, E). Total length 3.73. Carapace 1.59 long, 1.22 wide. Abdomen 1.91 long, 1.35 wide. Clypeus 0.08 high. Eye sizes and inter-distances: AME 0.35, ALE 0.23, PLE 0.20, AERW 1.12, PERW 1.02, EFL 0.73. Legs: I 2.53 (0.78, 0.50, 0.55, 0.40, 0.30), II 2.43 (0.75, 0.48, 0.50, 0.40, 0.30), III 3.04 (1.00, 0.50, 0.58, 0.63, 0.33), IV 2.99 (0.90, 0.43, 0.65, 0.68, 0.33). Habitus (Fig. 4E) similar to that of male except darker, and with a more slender body. Epigyne (Fig. 4A, B). Wider than long, with pair of small, posterolateral hoods; copulatory openings anteriorly located, almost round, separated from each other ca. their diameter; copulatory ducts thick, strongly curved ~ 180° medially; spermathecae almost elliptical, separated from each other more than their width; fertilization ducts originate from the inner of anterior portions of spermathecae, extending almost transversely.

##### Distribution.

Known only from the type locality in Hainan Province.

### ﻿Genus *Heliophanoides* Prószyński, 1992

#### ﻿ Heliophanoides moi sp. nov.

Taxon classificationAnimaliaAraneaeSalticidae

BC846A97-C36F-51D4-9EB5-A627B907164D

https://zoobank.org/80FFFF68-D152-436A-B22A-0836D17E227C

Fig. 5

##### Type material.

***Holotype*** ♂ (IZCAS-Ar44502), China: Hainan: Ledong County, Jianfengling National Nature Reserve, Wufenqu (18°44.03′N, 108°55.46′E, ca. 960 m), 15.viii.2010, G. Zheng leg. ***Paratypes*** 1♂ (TRU-JS 0686), Jianfengling National Nature Reserve, Peak Mountain (18°43.11′N, 108°52.32′E, ca. 1400 m), 22.iii.2023, Yunhu Mo leg.; 2♂ (IZCAS-Ar44503–44504), Lingshui County, Diaoluoshan National Nature Reserve, Direction of the Mysterious Tree (18°43.50′N, 108°52.10′E, ca. 920 m), 18.iv.2011, Y.Y. Zhou leg.

##### Etymology.

The specific name is after Mr. Yunhu Mo, one of the collectors of the type specimens; noun (name) in genitive case.

##### Diagnosis.

**Figure 5.**
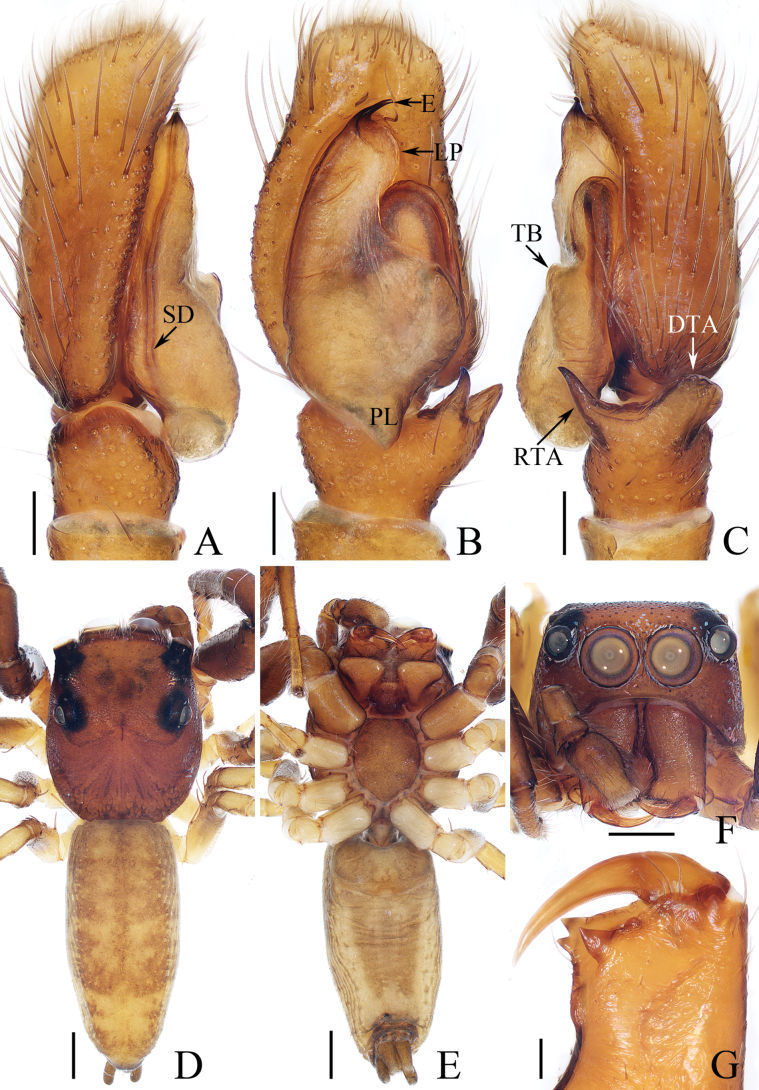
Male of *Heliophanoidesmoi* sp. nov., holotype **A** palp, prolateral **B** ditto, ventral **C** ditto, retrolateral **D** habitus, dorsal **E** ditto, ventral **F** carapace, frontal **G** chelicera, posterior. Scale bars: 0.1 mm (**A–C, G**); 0.5 mm (**D–F**).

*Heliophanoidesmoi* sp. nov. resembles that of *Phintellatengchongensis* Lei & Peng, 2013 in having similar palp structure, but it can be easily distinguished by the RTA, which is extending anteroventrally, and slightly less than tibia length in retrolateral view (Fig. 5C), vs. extending anteriorly, and ~ 1.5× longer than tibia in *P.tengchongensis* (Lei and Peng 2013: fig. 8b). It also somewhat resembles that of *Echinussaimerinensis* Simon, 1901 in the general shape of palpal structure, but it can be easily distinguished by the tapered RTA, which does not extend ventrally beyond the bulb prolateral margin in retrolateral view (Fig. 5C), vs. the RTA narrowest medio-posteriorly, and extends beyond the bulb prolateral margin in *E.imerinensis* (see the figure in Prószyński 1987).

##### Description.

**Male** (Fig. 5). Total length 4.70. Carapace 2.03 long, 1.73 wide. Abdomen 2.70 long, 1.24 wide. Clypeus 0.10 high. Eye sizes and inter-distances: AME 0.49, ALE 0.27, PLE 0.24, AERW 1.57, PERW 1.51, EFL 0.92. Legs: I 5.96 (1.80, 1.00, 1.58, 1.03, 0.55), II 3.70 (1.13, 0.58, 0.88, 0.68, 0.43), III 4.19 (1.30, 0.63, 0.73, 0.98, 0.55), IV 4.81 (1.40, 0.60, 1.13, 1.15, 053). Carapace red-brown, covered with sparse colourful scale-like setae and thin setae; fovea dark red, longitudinal, linear. Chelicerae red-brown, each with two promarginal teeth and one retromarginal tooth. Endites paler than chelicerae and widened distally. Labium red-brown, bearing brown setae at anterior margin. Sternum slightly longer than wide, with straight anterior margin. Legs pale yellow to red-brown, with strongest legs I bearing three and two pairs of ventral spines on the tibiae and metatarsi. Abdomen elongated, dorsum yellow to brown, with alternate yellow and brown transverse bands, covered wholly by scutum; venter pale, with longitudinal, broad, brown band medially. Palp (Fig. 5A–C). Tibia slightly wider than long in ventral view; RTA slightly less than tibia length, extending anteroventrally, tapered to pointed tip in retrolateral view; DTA flat, almost triangular in ventral view, almost square, and extending anterodorsally in retrolateral view; cymbium ~ 2× longer than wide, setose; bulb elongated, with sub-triangular posterior lobe, medio-retrolaterally located, lamellar tegular bump; embolus strongly sclerotized, short, curved retrolaterally with blunt tip, accompanied by lamellar process more than four times longer than wide, and with arc-shaped out-margin.

**Female.** Unknown.

##### Distribution.

Known only from the type locality in Hainan, China.

### ﻿Genus *Indopadilla* Caleb & Sankaran, 2019

#### ﻿ Indopadilla songi sp. nov.

Taxon classificationAnimaliaAraneaeSalticidae

6BC8959C-AF3A-5BD2-A610-35B5EC4E380F

https://zoobank.org/197A205C-137D-478F-80A0-1D5615731DE7

Figs 6 , 7

##### Type material.

***Holotype*** ♂ (TRU-JS 0687), CHINA: Hainan: Ledong County, Jianfengling National Nature Reserve, Peak Mountain (18°43.11′N, 108°52.32′E, ca. 1400 m), 17.iv.2019, C. Wang & Y.F. Yang leg. ***Paratypes*** 2♂1♀ (TRU-JS 0688–0690), same data as holotype; 1♂ (IZCAS-Ar44505), Jianfengling National Nature Reserve, Wufenqu (18°44.42′N, 108°51.80′E, ca. 800 m), 18.v.2011, Y.Y. Zhou leg.

##### Etymology.

The specific name is after the late Prof. Daxiang Song (1935–2008), who has made significant contributions to the taxonomy of Hainan jumping spiders; noun (name) in genitive case.

##### Diagnosis.

**Figure 6.**
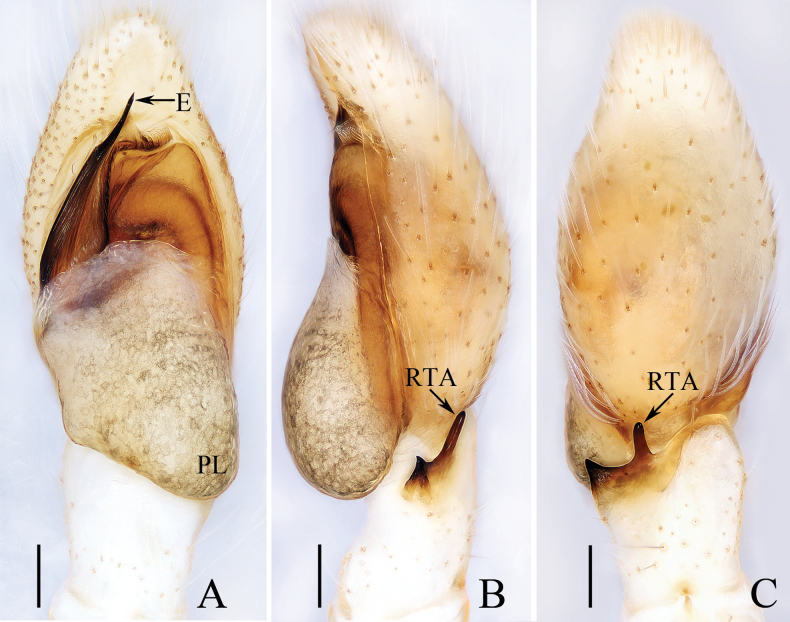
Male palp of *Indopadillasongi* sp. nov., holotype **A** ventral **B** retrolateral **C** dorsal. Scale bars: 0.1 mm.

**Figure 7.**
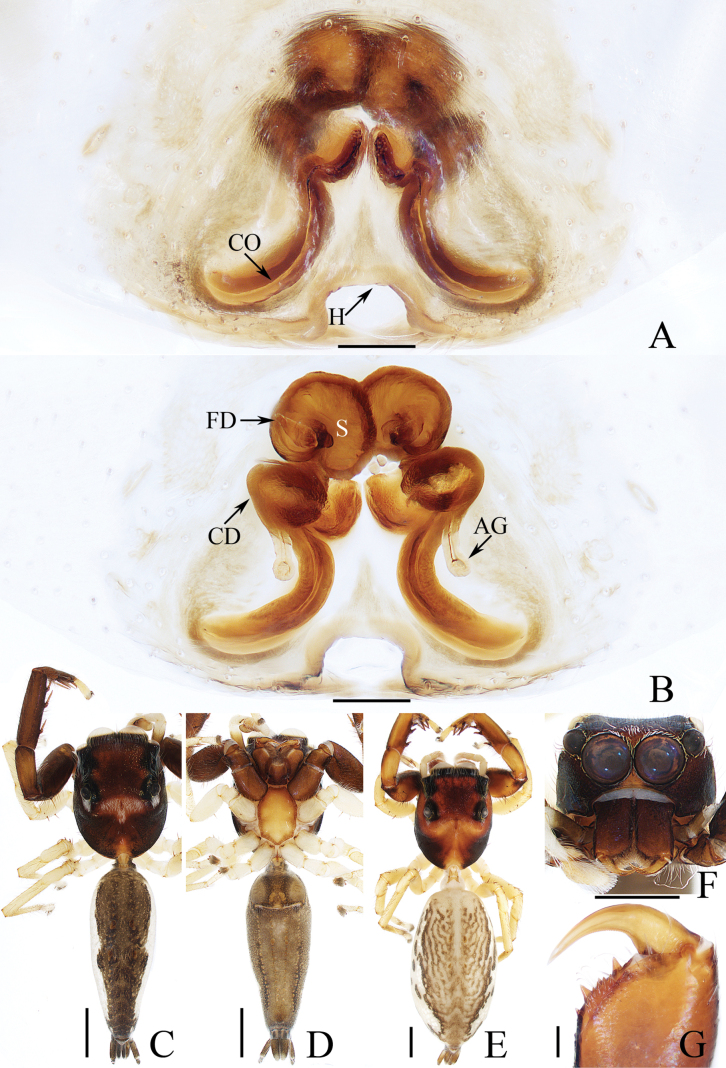
*Indopadillasongi* sp. nov., male holotype and female paratype **A** epigyne, ventral **B** vulva, dorsal **C** holotype habitus, dorsal **D** ditto, ventral **E** female paratype habitus, dorsal **F** holotype carapace, frontal **G** holotype chelicera, posterior. Scale bars: 0.1 mm (**A, B, G**); 1.0 mm (**C–F**).

The male of *Indopadillasongi* sp. nov. resembles that of *I.sabivia* Maddison, 2020 in having the bifurcated RTA, but it can be easily distinguished by the RTA has bar-shaped dorsal ramus in retrolateral view (Fig. 6B), vs. tapered, almost triangular dorsal ramus in *I.sabivia* (Maddison et al. 2020: fig. 118). The female resembles that of *I.cuc* Wang, Li & Pham, 2023 in having similar epigyne, but it can be distinguished by the following: (1) the epigynal hood is almost square in ventral view (Fig. 7A), vs. almost half-round in *I.cuc* (Wang et al. 2023: fig. 14A); (2) the AG extends posteriorly, and has slightly enlarged terminus (Fig. 7B), vs. extending towards lateral sides, and without enlarged terminus in *I.cuc* (Wang et al. 2023: fig. 14B).

##### Description.

**Male** (Figs 6, 7C, D, F, G). Total length 6.05. Carapace 2.40 long, 1.95 wide. Abdomen 3.50 long, 1.40 wide. Clypeus 0.06 high. Eye sizes and inter-distances: AME 0.64, ALE 0.30, PLE 0.29, AERW 1.73, PERW 1.68, EFL 1.10. Legs: I 6.68 (1.95, 1.05, 1.75, 1.25, 0.68), II 4.74 (1.38, 0.75, 1.08, 0.98, 0.55), III 4.31 (1.30, 0.70, 0.83, 0.98, 0.50), IV 5.45 (1.65, 0.70, 1.20, 1.35, 0.55). Carapace dark red, with a pair of white setae behind the PLEs, and arc-shaped, red-orange area bearing dense white setae anteriorly on thorax; fovea red-brown, longitudinal. Chelicerae red-brown, each with four promarginal teeth and one retromarginal fissidental tooth with seven cusps. Endites longer than wide, bearing dense brown setae distally on the inner margins. Labium coloured as endites, bearing brown setae on anterior margin. Sternum yellow to red, ~ 1.5× longer than wide. Legs pale to red-brown, legs I strongest, with three and two pairs of ventral spines on tibiae and metatarsi. Abdomen elongated, dorsum green-brown except the lateral sides white, dotted, with two pairs of muscle depressions medially; venter paler than the dorsum, with four, longitudinal, dotted lines. Palp (Fig. 6A–C). Tibia longer than wide; RTA bifurcated, with strongly sclerotized, apically pointed ventral ramus and bar-shaped dorsal ramus; cymbium ~ 2× longer than wide, covered with pale setae; bulb longer than wide, swollen medio-posteriorly, with posterior lobe extending postero-retrolaterally; embolus long and broad, distally divided into the strongly sclerotized, needle-shaped portion, and the weakly sclerotized, irregular portion.

**Female** (Fig. 7A, B, E). Total length 9.01. Carapace 3.16 long, 2.64 wide. Abdomen 5.19 long, 2.92 wide. Clypeus 0.08 high. Eye sizes and inter-distances: AME 0.78, ALE 0.43, PLE 0.40, AERW 2.26, PERW 2.17, EFL 1.51. Legs: I 7.44 (2.25, 1.38, 1.88, 1.30, 0.63), II 5.91 (1.80, 1.05, 1.38, 1.05, 0.63), III 5.51 (1.75, 0.90, 0.98, 1.25, 0.63), IV 7.14 (2.13, 0.95, 1.68, 1.75, 0.63). Habitus (Fig. 7E) similar to that of male, except paler and with one retromarginal fissidental tooth with ten cusps. Epigyne (Fig. 7A, B). Slightly longer than wide, with sub-square, posterior hood; copulatory openings slit-shaped; copulatory ducts short, swollen, with posteriorly extending accessory glands forming round terminus; spermathecae almost oval, partly overlapped.

##### Distribution.

Known only from the type locality in Hainan Island, China.

#### ﻿Genus Logunattus gen. nov.

Taxon classificationAnimaliaAraneaeSalticidae

F65EF1A6-99AD-5730-9378-A47E4B48DE07

https://zoobank.org/85B9CF3E-9432-4CCE-B30B-9F27A886BF2E

##### Type species.

*Logunattuslibaii* sp. nov. from Hainan, China designated herein.

##### Etymology.

The specific name is a combination of *logun*, referring to Dr. Dmitri V. Logunov (Manchester, UK), a leading arachnologist in jumping spiders, and *attus*, meaning jumper. The gender is masculine.

##### Diagnosis.

*Logunattus* gen. nov. can be recognized as a member of the tribe Euophryini Simon, 1901 by the similarity of habitus and palpal structure to the representative genus of this tribe, *Euochin* Prószyński, 2018, especially the presence of white setae on the dorsum of palpal tibia and cymbium and the loop of sperm duct inside the tegulum (Maddison 2015). It can be easily recognized by the dagger-axe-shaped RTA. It resembles that of *Euochin* Prószyński, 2018 in having similar habitus, the presence of white setae on the dorsum of tibia and cymbium of male palp, and large spermathecae, but it can be distinguished by the following: (1) the embolus is straight or curved, vs. coiled in *Euochin* (Zha et al. 2014: figs 5, 8, 16, 19; Metzner 2023); (2) the RTA is dagger-axe-shaped in retrolateral view, vs. straight in *Euochin* (Zha et al. 2014: figs 6, 9, 17, 20; Metzner 2023); (3) the chelicera has a single retrolateral tooth, vs. a retromarginal fissidental tooth with several cusps in the generotype and its congeners of *Euochin* (see the description in Zha et al. 2014); (4) the epigyne has pair of non-transparent atria lack ridges, vs. transparent atria have concomitant lateral ridges in *Euochin* (Zha et al. 2014: figs 3, 10, 14, 21; Metzner 2023). The genus also somewhat resembles that of *Spiralembolus* gen. nov. in having similar habitus and copulatory organs, but it can be easily distinguished by the absence of white setae on clypeus, the dagger-axe-shaped RTA, non-spiralled embolus, the presence of median septum, and accessory glands of copulatory ducts, vs. presence of a cluster of white setae on clypeus, RTA non-dagger-axe-shaped, spiralled embolus, the absence of median septum and accessory glands of copulatory ducts in *Spiralembolus* (Figs 19, 20A–C, G, 21, 22A–C, G).

**Figure 8.**
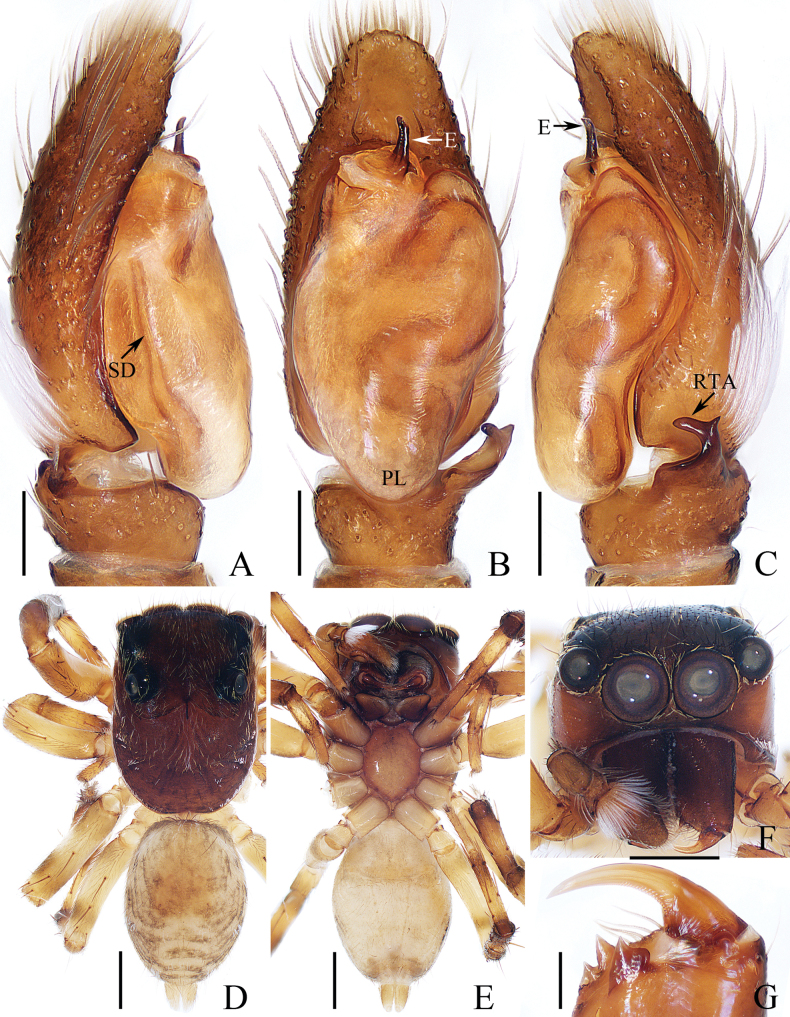
Male of *Logunattusdufui* sp. nov., holotype **A** palp, prolateral **B** ditto, ventral **C** ditto, retrolateral **D** habitus, dorsal **E** ditto, ventral **F** carapace, frontal **G** chelicera, posterior. Scale bars: 0.1 mm (**A–C, G**); 0.5 mm (**D–F**).

**Figure 9.**
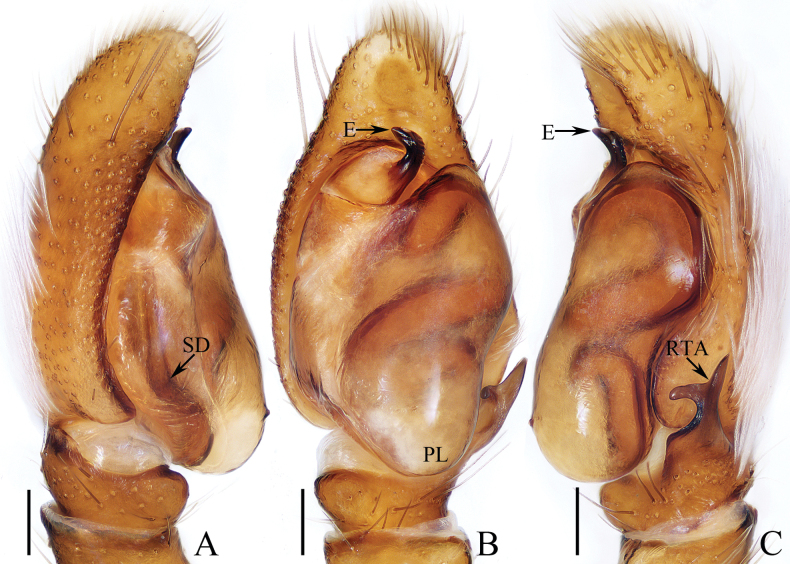
Male palp of *Logunattuslibaii* sp. nov., holotype **A** prolateral **B** ventral **C** retrolateral. Scale bars: 0.1 mm.

##### Description.

Small-sized jumping spider. Sexual dimorphism indistinct. Carapace sub-square, covered with dense white and yellow setae, with elevated cephalic region and sloped thorax; fovea longitudinal, dark, linear. Chelicerae yellow-brown, each with two promarginal teeth and one retromarginal tooth. Endites paler than chelicerae, broadened mediodistally. Labium almost linguiform, paler anteriorly. Sternum longer than wide, almost heart-shaped, with straight anterior margin. Legs yellow to dark brown, covered with sparse spines. Abdomen sub-oval, the dorsum yellow to grey-brown, with small anteromedian scutum that only presented in males; venter paler than dorsum, with pair of longitudinal, central dotted lines.

Palp. Tibia short, with ventro-prolateral bump, dagger-axe-shaped RTA, and dense, white setae dorsally; cymbium longer than wide, with dense white setae dorsally on proximal half; bulb elongate-oval, with distinct posterior lobe; embolus originates from antero-prolateral portion of bulb, forming disc at base, and curved or straight medially, with spinous base apophysis or not.

Epigyne. See the description of the generotype.

##### Composition.

The genus includes two species, the generotype, and *G.dufui* sp. nov.

##### Distribution.

Known only from Hainan Island, China.

#### ﻿ Logunattus dufui sp. nov.

Taxon classificationAnimaliaAraneaeSalticidae

08EC9FCD-0481-57A4-933E-6B35DE5A8B80

https://zoobank.org/26D1252B-58C8-4507-8F2C-8E2D143C77E8

Fig. 8

##### Type material.

***Holotype*** ♂ (IZCAS-Ar44506), China: Hainan: Lingshui County, Diaoluoshan Resort (18°43.51′N, 108°52.10′E, ca. 920 m), 20.iv.2011, Y.Y. Zhou leg. ***Paratypes*** 3♂ (IZCAS-Ar44507–44509), same data as holotype.

##### Etymology.

The specific name is after Mr. Fu Du (712–770), a famous ancient Chinese poet, who was crowned as Poetry Sage; noun (name) in genitive case.

##### Diagnosis.

*Logunattusdufui* sp. nov. resembles that of *L.libaii* sp. nov. in having similar habitus and palpal structure, but it can be easily distinguished by the embolus, which is straight and with spinous proximal apophysis (Fig. 8B), vs. curved and without apophysis in *L.libaii* (Fig. 9B).

##### Description.

**Male** (Fig. 8). Total length 3.32. Carapace 1.79 long, 1.28 wide. Abdomen 1.47 long, 1.10 wide. Clypeus 0.05 high. Eye sizes and inter-distances: AME 0.38, ALE 0.26, PLE 0.20, AERW 1.22, PERW 1.10, EFL 0.76. Legs: I 4.08 (1.19, 0.68, 1.00, 0.73, 0.48), II 2.93 (0.93, 0.50, 0.60, 0.55, 0.35), III 3.48 (1.13, 0.45, 0.75, 0.75, 0.40), IV 3.59 (1.08, 0.50, 0.75, 0.83, 0.43). Carapace red-brown, covered with yellow setae on face and white setae on thorax; fovea dark, longitudinal, linear. Chelicerae each with two promarginal teeth and one retromarginal tooth. Endites broadened medio-distally. Labium linguiform. Sternum longer than wide, with straight anterior margin. Legs yellow to brown, with three and two pairs of ventral spines on tibiae and metatarsi I and II, respectively. Abdomen suboval, dorsum pale to brown, covered with sparse white and brown setae, with two pairs of anterior muscle depressions and anteromedian scutum ca. half the abdominal width and length; venter yellow to pale yellow, with pair of longitudinal, dotted lines medially. Palp (Fig. 8A–C, E). Tibia very short, with ventro-prolateral bump, and dagger-axe-shaped RTA; cymbium ~ 2× longer than wide in ventral view, covered with dense white setae dorso-proximally; bulb elongated, with tapered, downward extending posterior lobe; embolus short and straight, forming disc and with spinous apophysis at base, and blunt apically.

**Female.** Unknown.

##### Distribution.

Known only from the type locality in Hainan Island, China.

#### ﻿ Logunattus libaii sp. nov.

Taxon classificationAnimaliaAraneaeSalticidae

A8B2CE66-1B9B-56DD-AC5D-DB0237E7B316

https://zoobank.org/F8108ED6-FEE9-4F00-9709-8377ABC716E2

Figs 9 , 10

##### Type material.

***Holotype*** ♂ (IZCAS-Ar44510), China: Hainan: Qiongzhong County, Limushan National Nature Reserve, Diaodengling Waterfall (19°10.87′N, 109°45.32′E, ca. 940 m), 5.v.2011, Y.Y. Zhou leg. ***Paratypes*** 1♂2♀ (IZCAS-Ar44511–44513), same data as holotype; 2♀ (IZCAS-Ar44514–44515), Limushan National Nature Reserve, Direction of Shuiba (19°10.87′N, 109°45.32′E, ca. 960 m), 5.v.2011, Y.Y. Zhou leg.; 1♂2♀ (IZCAS-Ar44516–44518), Wuzhishan City, Wuzhishan National Nature Reserve, Shanye Hotel (18°54.42′N, 109°40.65′E, ca. 1590 m), 26.iv.2011, Y.Y. Zhou leg.

##### Etymology.

The specific name is after Mr. Bai Li (701–762), a famous ancient Chinese poet, who was crowned as Poetry Immortal; noun (name) in genitive case.

##### Diagnosis.

**Figure 10.**
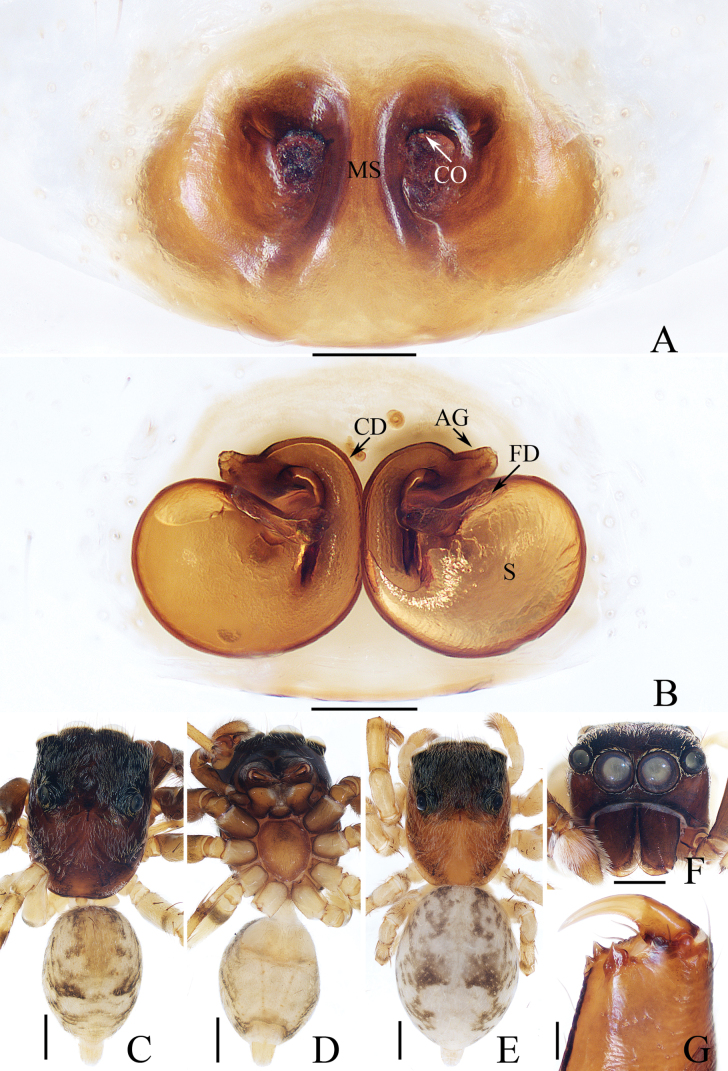
*Logunattuslibaii* sp. nov., male holotype and female paratype **A** epigyne, ventral **B** vulva, dorsal **C** holotype habitus, dorsal **D** ditto, ventral **E** female paratype habitus, dorsal **F** holotype carapace, frontal **G** holotype chelicera, posterior. Scale bars: 0.1 mm (**A, B, G**); 0.5 mm (**C–F**).

The male of *Logunattuslibaii* sp. nov. resembles that of *L.dufui* sp. nov. in general shape of palp, but it can be easily distinguished by the embolus, which is curved medially, and without proximal apophysis in ventral view (Fig. 9B), vs. straight and with proximal apophysis in *L.dufui* (Fig. 8B). The female of this new species resembles that of *Spiralembolusyinggeling* sp. nov. in having similar epigyne, but it can be easily distinguished by the presence of median septum, and accessory glands of copulatory ducts (Fig. 10A, B), vs. absent in *S.yinggeling* (Fig. 20A, B).

##### Description.

**Male** (Figs 9, 10C, D, F, G). Total length 3.45. Carapace 1.83 long, 1.37 wide. Abdomen 1.53 long, 1.15 wide. Clypeus 0.08 high. Eye sizes and inter-distances: AME 0.38, ALE 0.26, PLE 0.20, AERW 1.32, PERW 1.20, EFL 0.79. Legs: I 4.08 (1.15, 0.68, 1.05, 0.75, 0.45), II 3.16 (0.93, 0.58, 0.70, 0.55, 0.40), III 3.77 (1.15, 0.58, 0.86, 0.78, 0.40), IV 3.79 (1.10, 0.53, 0.83, 0.95, 0.38). Carapace red-brown to dark brown, covered with dense setae; fovea dark, longitudinal, bar-shaped. Chelicerae red-brown to dark brown, each with two promarginal teeth and one retromarginal tooth. Endites dark yellow, with pale distal-inner margins bearing dense brown setae. Labium darker than endites. Sternum coloured as endites, slightly longer than wide, with straight anterior margin, widest medially. Legs yellow to dark brown, with three and two pairs of ventral spines on tibiae and metatarsi I and II, respectively. Abdomen oval, dorsum with two pairs of anterior muscle depressions, indistinct brown or dark brown stripes, and big, irregular pale marking posteriorly, covered by anteromedian scutum; venter pale, with pair of longitudinal, dotted lines. Palp (Fig. 9A–C). Tibia short, with ventro-prolateral bump, covered with white dorsal setae; RTA dagger-axe-shaped, almost 1.5 times longer than tibia, with pointed tip; cymbium ~ 1.8× longer than wide in ventral view, covered with dorsal white setae at proximal half; bulb elongate-oval, with blunt posterior lobe extending postero-retrolaterally; embolus originates from the antero-prolateral portion of bulb, forming a disc at base, curved towards prolateral side medially and with rather pointed tip directed towards ~ 10:30 o’clock position.

**Female** (Fig. 10A, B, E). Total length 4.02. Carapace 1.87 long, 1.39 wide. Abdomen 2.18 long, 1.64 wide. Clypeus 0.09 high. Eye sizes and inter-distances: AME 0.40, ALE 0.27, PLE 0.20, AERW 1.26, PERW 1.15, EFL 0.84. Legs: I 3.59 (1.05, 0.63, 0.88, 0.60, 0.43), II 3.14 (0.98, 0.53, 0.70, 0.50, 0.43), III 3.89 (1.23, 0.60, 0.85, 0.78, 0.43), IV 3.99 (1.18, 0.53, 0.88, 0.95, 0.45). Habitus (Fig. 10E) similar to that of male except paler, and without scutum on the dorsum of abdomen. Epigyne (Fig. 10A, B). Wider than long, with pair of shallow atria anteromedially; copulatory openings almost half round, open towards downward, separated by the slightly raised median septum; copulatory ducts curved medially, and touched distally, with sub-triangular proximal accessory glands extending antero-prolaterally; spermathecae oval; fertilization ducts lamellar, extending transversely.

##### Distribution.

Known only from the type locality in Hainan Island, China.

### ﻿Genus *Myrmarachne* MacLeay, 1839

#### ﻿ Myrmarachne mixiaoqii sp. nov.

Taxon classificationAnimaliaAraneaeSalticidae

7C6F680E-98D6-51EE-A4E4-B0D9281A317B

https://zoobank.org/88020DA0-B47B-44A5-A816-C79E045C8073

Figs 11 , 12

##### Type material.

***Holotype*** ♂ (IZCAS-Ar44519), China: Hainan: Wuzhishan City, Wuzhishan National Nature Reserve, hillside (18°53.83′N, 109°41.88′E, ca. 1590 m), 9.iv.2009, G. Tang leg. ***Paratypes*** 2♀ (IZCAS-Ar44520–44521), same data as holotype; 1♀ (IZCAS-Ar44522), same site as holotype, 8.iv.2009, G. Tang leg.; 1♀ (IZCAS-Ar44523), Lingshui County, Diaoluoshan National Nature Reserve, Power Station (18°39.84′N, 109°55.81′E, ca. 100 m), 20.iv.2009, G. Tang leg.

##### Etymology.

The specific name is a patronym of Prof. Xiaoqi Mi, who greatly helped us with this research; noun (name) in genitive case.

##### Diagnosis.

*Myrmarachnemixiaoqii* sp. nov. can be easily distinguished from other congeners by the presence of a cluster of cymbial macro-setae above the dRTA, and the pair of bag-shaped structures below the epigynal hood, vs. the absence of a cluster of cymbial macro-setae above the dRTA and without similar bag-shaped structures below the epigynal hood in congeners presently known (see Metzner 2023).

##### Description.

**Figure 11.**
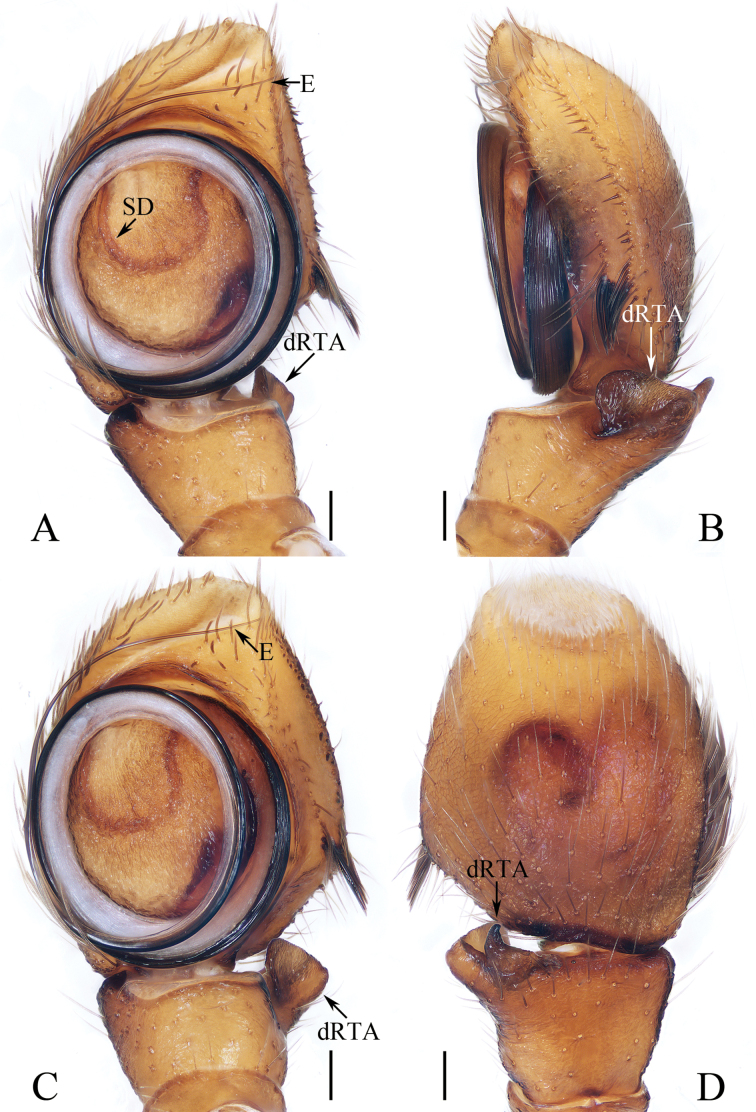
Male palp of *Myrmarachnemixiaoqii* sp. nov., holotype **A, C** ventral **B** retrolateral **D** dorsal. Scale bars: 0.1 mm.

**Figure 12.**
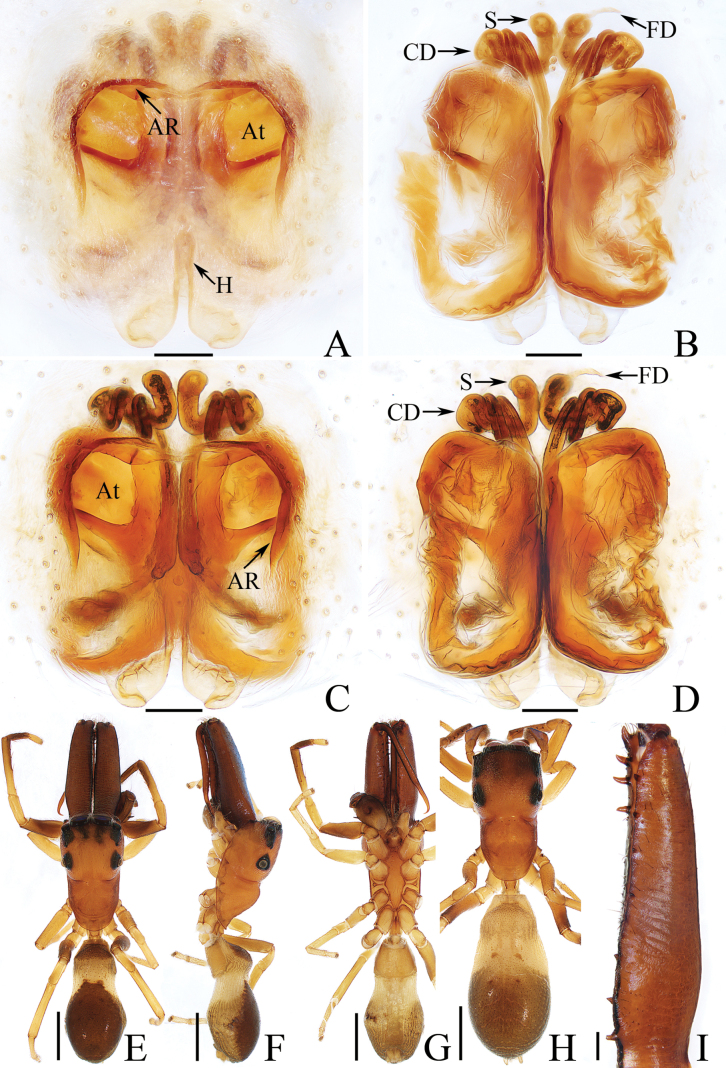
*Myrmarachnemixiaoqii* sp. nov., male holotype and female paratype **A, C** epigyne, ventral **B, D** vulva, dorsal **E** holotype habitus, dorsal **F** ditto, lateral **G** ditto, ventral **H** female paratype habitus, dorsal **I** holotype chelicera, posterior. Scale bars: 0.1 mm (**A–D**); 0.2 mm (**I**); 1.0 mm (**E–H**).

**Male** (Figs 11, 12E–G, I). Total length 5.02. Carapace 2.25 long, 1.34 wide. Abdomen 2.60 long, 1.23 wide. Clypeus 0.09 high. Eye sizes and inter-distances: AME 0.43, ALE 0.22, PLE 0.20, AERW 1.26, PERW 1.30, EFL 0.94. Legs: I 4.47 (1.33, 0.68, 1.30, 0.73, 0.43), II 3.29 (1.00, 0.58, 0.83, 0.55, 0.33), III 3.38 (1.00, 0.50, 0.80, 0.75, 0.33), IV 4.71 (1.40, 0.63, 1.25, 1.08, 0.35). Carapace dark yellow, with elevated cephalic region bearing pair of irregular dark patches behind AMEs, and medially raised thorax. Chelicerae elongated, longer than carapace, each with four promarginal and eight retromarginal teeth. Endites yellow, elongated. Labium slightly darker than endites. Sternum narrow, ~ 3× longer than wide. Legs yellow to brown, with one ventral spine on patellae I, four and two pairs of ventral spines on tibiae I and metatarsi I, respectively. Abdomen elongated, almost gourd-shaped, constricted at anterior 1/3, dorsum yellow to red-brown, covered wholly by scutum; venter pale to brown. Palp (Fig. 11A–D). Tibia wider than long in ventral view; dRTA bifurcated, with platelike ventral ramus and short, digitiform dorsal ramus; cymbium almost oval, bearing cluster of medio-retrolateral macro-setae above the dRTA; bulb almost round, flat, embolus long, coiled more than two coils, with pointed tip reaches the cymbial tip.

**Female** (Fig. 12A–D, H). Total length 6.00. Carapace 2.46 long, 1.35 wide. Abdomen 3.08 long, 1.58 wide. Clypeus 0.10 high. Eye sizes and inter-distances: AME 0.44, ALE 0.22, PLE 0.20, AERW 1.27, PERW 1.35, EFL 1.01. Legs: I 4.09 (1.25, 0.63, 1.18, 0.60, 0.43), II 3.17 (0.98, 0.53, 0.80, 0.53, 0.33), III 3.47 (1.00, 0.48, 0.83, 0.78, 0.38), IV 4.97 (1.48, 0.63, 1.33, 1.13, 0.40). Habitus (Fig. 12H) similar to that of male except with much shorter chelicerae each with five promarginal and nine or ten retromarginal teeth, without dorsal scutum on abdomen, and with six pairs of ventral spines on tibiae I. Epigyne (Fig. 12A–D). Longer than wide, with tube-shaped epigynal hood fused with pair of bag-shaped structures posteriorly; atria paired, with invert L-shaped lateral ridges; copulatory ducts long, forming complex paths with three distal coils; spermathecae almost spherical, anteriorly located, separated from each other slightly less than their diameter.

##### Distribution.

Only known from the type locality in Hainan Island, China.

### ﻿Genus *Nandicius* Prószyński, 2016

#### ﻿ Nandicius shihaitaoi sp. nov.

Taxon classificationAnimaliaAraneaeSalticidae

F9A856FB-9E10-5774-AEA2-D247DFB7C6E4

https://zoobank.org/B194D6DD-0B4C-4E0C-85A6-9F4ACF2BAB06

Figs 13 , 14

##### Type material.

***Holotype*** ♂ (IZCAS-Ar44524), China: Hainan: Baisha County, Yinggeling National Nature Reserve (19°03.05′N, 109°33.78′E, ca. 680 m), 21.viii.2010, G. Zheng leg. ***Paratypes*** 1♂1♀ (IZCAS–Ar44525–44526), same data as holotype; 1♀ (IZCAS-Ar44527), Lingshui County, Diaoluoshan National Nature Reserve, Plank Road (18°43.60′N, 109°51.99′E, ca. 950 m), 8.viii.2010, G. Zheng leg.; 1♀ (IZCAS-Ar44528), Jianfengling National Nature Reserve, Mingfeng Valley (18°44.65′N, 108°50.44′E, ca 1000 m), 18.viii.2010, G. Zheng Leg.

##### Etymology.

The specific name is after Prof. Haitao Shi, a leading scientist in turtle conservation; noun (name) in genitive case.

##### Diagnosis.

**Figure 13.**
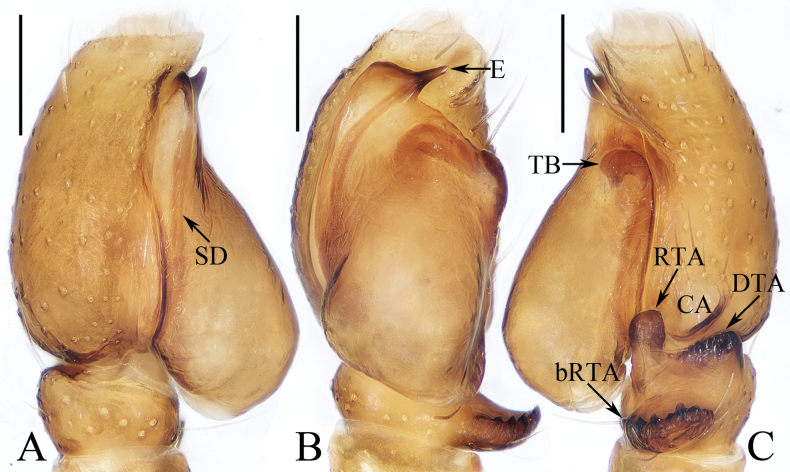
Male palp of *Nandiciusshihaitaoi* sp. nov., holotype **A** prolateral **B** ventral **C** retrolateral. Scale bars: 0.1 mm.

**Figure 14.**
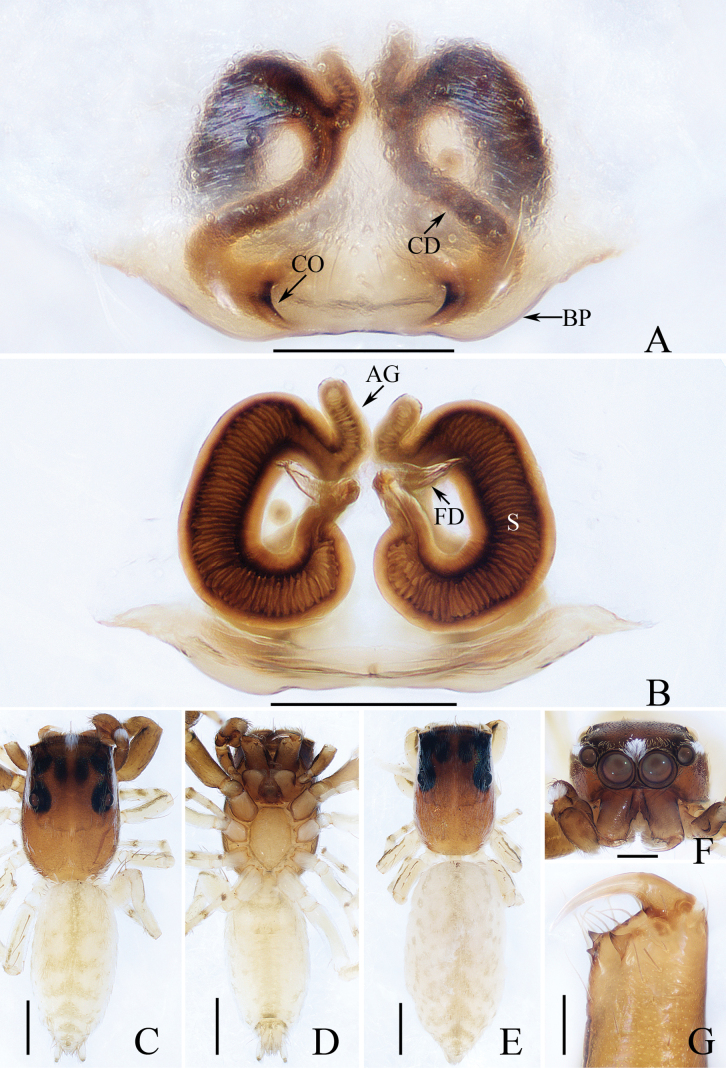
*Nandiciusshihaitaoi* sp. nov., male holotype and female paratype **A** epigyne, ventral **B** vulva, dorsal **C** holotype habitus, dorsal **D** ditto, ventral **E** female paratype habitus, dorsal **F** holotype carapace, frontal **G** holotype chelicera, posterior. Scale bars: 0.1 mm (**A, B, G**); 0.2 mm (**F**); 0.5 mm (**C–E**).

The male of *Nandiciusshihaitaoi* sp. nov. resembles that of *N.proszynskii* Wang & Li, 2021 in having very similar habitus and palpal structure, but it can be easily distinguished by the presence of bRTA (Fig. 13B, C), vs. bRTA absent in *N.proszynskii* (Wang and Li 2021: fig. 10B, C). The female closely resembles that of *Tasakoreana* (Wesołowska, 1981) in having very similar epigyne, but it can be easily distinguished by the presence of a basal epigynal plate, and the C-shaped copulatory openings (Fig. 14A), vs. the absence of basal epigynal plate and oval copulatory opeings in *T.koreana* (Suguro and Yahata 2014: fig. 29). The female also resembles that of *Madhyattusjabalpurensis* Prószyński, 1992 in having a very similar epigyne, but it can be easily distinguished by the distance between the copulatory openings, which is more than half the epigynal width (Fig. 14A), vs. ~ 1/3 the epigynal width in *M.jabalpurensis* (Prószyński 1992: fig. 77).

##### Description.

**Male** (Figs 13, 14C, D, F, G). Total length 2.87. Carapace 1.34 long, 0.92 wide. Abdomen 1.55 long, 0.81 wide. Clypeus 0.09 high. Eye sizes and inter-distances: AME 0.22, ALE 0.12, PLE 0.12, AERW 0.77, PERW 0.77, EFL 0.58. Legs: I 1.91 (0.58, 0.38, 0.45, 0.30, 0.20), II 1.46 (0.45, 0.30, 0.28, 0.25, 0.18), III 1.55 (0.48, 0.23, 0.33, 0.28, 0.23), IV 2.11 (0.73, 0.25, 0.50, 0.38, 0.25). Carapace yellow except the lateral sides of eye field black, covered with white and brown setae, with cluster of white setae at the median of anterior margin, pair of white stripes of setae laterally on cephalic region, and pair of elongate-oval dark spots medially on eye field; fovea indistinct. Chelicerae dark yellow, each with two promarginal teeth and one retromarginal tooth. Endites longer than wide, widened distally, bearing dense setae distally on inner margins. Labium tapered, almost linguiform. Sternum ~ 1.5× longer than wide, with straight anterior margin. Legs pale to dark yellow, with green-brown stripes on the lateral of femora, patellae, and metatarsi II, III, IV; leg I strongest, with slightly enlarged femora and tibiae. Abdomen elongated, dorsum with several transverse chevron patterns medio-posteriorly; venter pale. Palp (Fig. 13A–C). Tibia wider than long, with three apophyses, including the retrolateral one curved inwards distally and blunt apically, the dorsal one almost triangular, and the broad baso-retrolateral one with several small apophyses; cymbium ~ 1.5× longer than wide, with tuberous proximal apophysis; bulb elongated, swollen medio-posteriorly, with antero-retrolateral bump; embolus originates from the antero-prolateral portion of bulb, short and straight, tapered to the rather pointed tip directed towards ~ 2 o’clock position.

**Female** (Fig. 14A, B, E). Total length 3.18. Carapace 1.22 long, 0.80 wide. Abdomen 1.90 long, 1.02 wide. Clypeus 0.10 high. Eye sizes and inter-distances: AME 0.23, ALE 0.13, PLE 0.12, AERW 0.72, PERW 0.75, EFL 0.54. Legs: I 1.54 (0.48, 0.30, 0.33, 0.25, 0.18), II 1.34 (0.43, 0.25, 0.28, 0.20, 0.18), III 1.52 (0.48, 0.23, 0.30, 0.28, 0.23), IV 2.11 (0.70, 0.28, 0.50, 0.38, 0.25). Habitus (Fig. 14E) similar to that of male except without the cluster of setae on the median of the anterior margin of carapace. Epigyne (Fig. 14A, B). Slightly wider than long, with arc-shaped base plate; copulatory openings posteriorly located, C-shaped, and separated from each other ca. half the epigynal width; copulatory ducts thickest proximally, curved into S-shape, with curved, bar-shaped, terminal accessory glands; spermathecae elongated, curved into C-shape.

##### Distribution.

Known only from the type locality in Hainan Island, China.

##### Comments.

The new species is placed into the genus provisionally because it shares a very similar habitus and palpal structure with the known congener, *Nandiciusproszynskii* Wang & Li, 2021. However, it is inconsistent with other congeners in the epigynal structure and body shape, which indicates its generic position needs further confirmation.

### ﻿Genus *Pancorius* Simon, 1902

#### ﻿ Pancorius hainanensis

Taxon classificationAnimaliaAraneaeSalticidae

Song & Chai, 1991

FAE3D068-3E8F-5EEA-AD33-45DEE4FAE233

Figs 15 , 16

Pancorius hainanensis Song & Chai, 1991: 20, fig. 10A, B (male holotype, examined).

##### Type material examined.

***Holotype*** ♂, China: Hainan: Bawangling National Nature Reserve, xii.1989, M.S. Zhu leg.

##### Other material examined.

1♂1♀ (TRU-JS 0691–0692), China: Hainan: Qiongzhong County, Limushan National Nature Reserve, 1–5.v.2021, F.E. Li leg.

##### Diagnosis.

**Figure 15.**
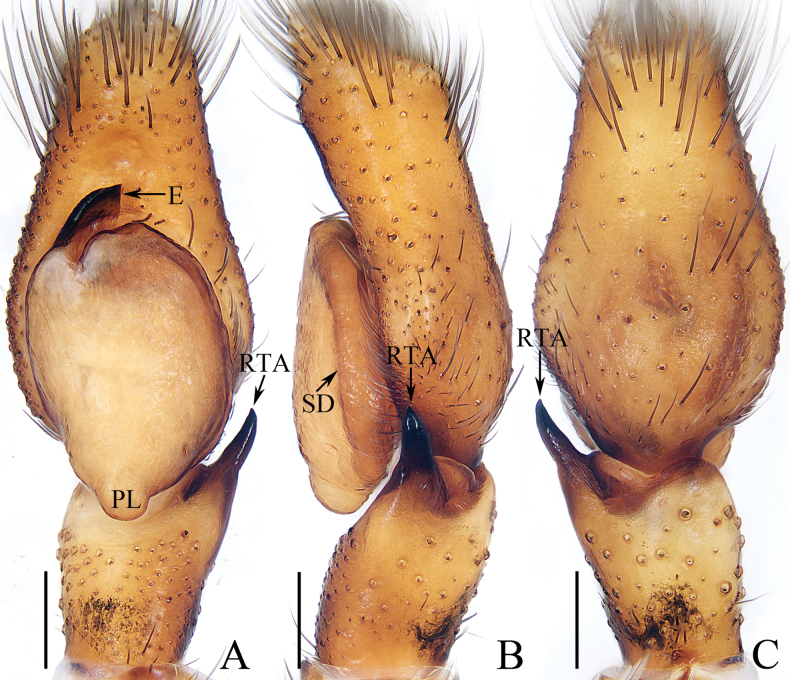
Male palp of *Pancoriushainanensis* Song & Chai, 1991 **A** ventral **B** retrolateral **C** dorsal. Scale bars: 0.2 mm.

**Figure 16.**
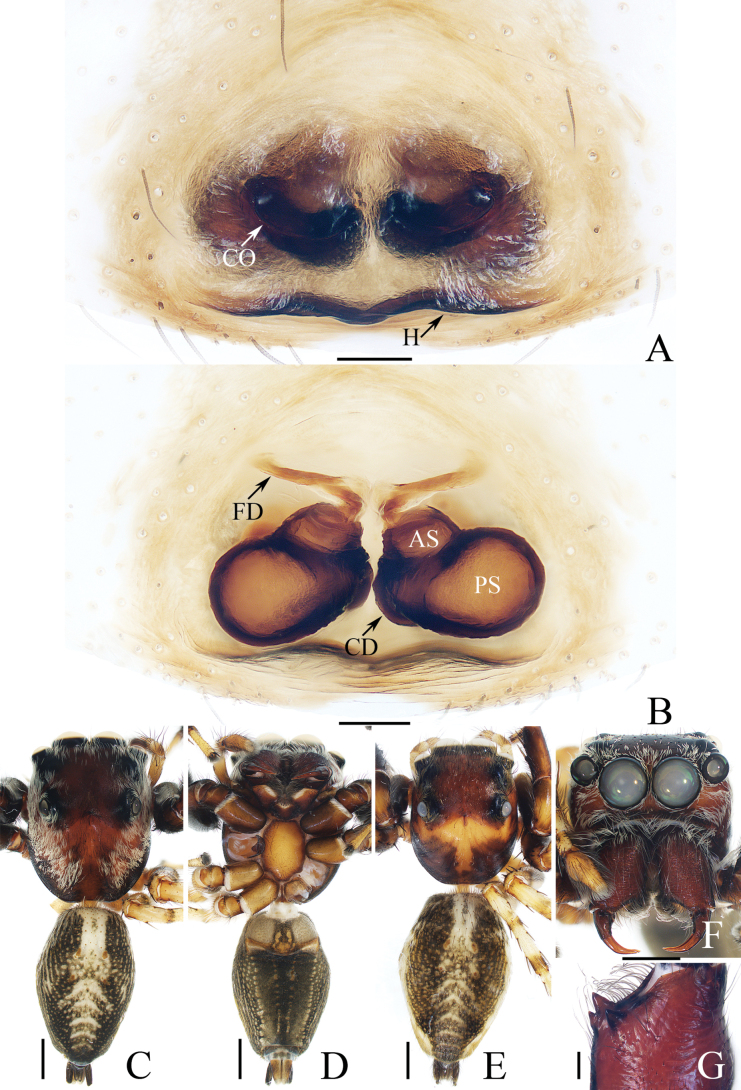
*Pancoriushainanensis* Song & Chai, 1991 **A** epigyne, ventral **B** vulva, dorsal **C** male habitus, dorsal **D** ditto, ventral **E** female habitus, dorsal **F** male carapace, frontal **G** male chelicera, posterior. Scale bars: 0.1 mm (**A, B, G**); 1.0 mm (**C–F**).

The male of *Pancoriushainanensis* Song & Chai, 1991 resembles that of *P.wesolowskae* Wang & Wang, 2020 in the general shape of palpal structure, especially the small, blunt posterior lobe, but it can be easily distinguished by the following: (1) the RTA being slightly greater than sperm duct diameter in width, and with a pointed tip in retrolateral view (Fig. 15B), vs. more than two times greater than sperm duct diameter in width, and blunt apically in *P.wesolowskae* (Wang and Wang 2020: fig. 20), (2) the embolus acutely narrowed to the pointed tip distally in ventral view (Fig. 15A) vs. almost tapered in *P.wesolowskae* (Wang and Wang 2020: fig. 19). The female of this species resembles that of *P.crinitus* Logunov & Jäger, 2015 in having very shallow epigynal hoods, but it can be easily distinguished by anterior chamber of spermathecae, which are wider than long (Fig. 16B), vs. ~ 2× longer than wide in *P.crinitus* (Logunov and Jäger 2015: fig. 43).

##### Description.

**Male** (Figs 15, 16C, D, F, G). Total length 8.21. Carapace 3.94 long, 3.16 wide. Abdomen 4.00 long, 2.47 wide. Clypeus 0.18 high. Eye sizes and inter-distances: AME 0.91, ALE 0.58, PLE 0.52, AERW 2.95, PERW 2.63, EFL 1.84. Legs: I 9.31 (2.58, 1.70, 2.45, 1.63, 0.95), II 8.21 (2.65, 1.45, 1.88, 1.35, 0.88), III 9.18 (2.98, 1.30, 2.10, 1.80, 1.00), IV 8.16 (2.50, 1.13, 1.80, 1.85, 0.88). Carapace red-brown to dark brown, covered with white and dark setae, dense on both sides; fovea longitudinal, dark. Chelicerae red-brown, each with two promarginal teeth and one retromarginal tooth. Endites paler than chelicerae, bearing dense dark brown setae on the distal half of inner margins. Labium tapered, with pale distal end. Sternum yellow to red-brown, shield-shaped, ~ 1.5× longer than wide. Legs yellow to dark, setose, and spiny. Abdomen elongated, dorsum dark brown and mingled with green, spotted, with longitudinal, pale band anteromedially, two pairs of median muscle depressions followed by four transverse arc-shaped pale stripes; venter dark brown, spotted laterally, with pair of longitudinal, dotted lines medially. Palp (Fig. 15A–C). Tibia longer than wide, with strongly sclerotized RTA slightly curved inward distally and pointed apically; cymbium 1.5× longer than wide; bulb slightly swollen, with small bean-shaped posterior lobe; embolus short, broad, and flat, slightly curved retrolaterally, with pointed tip.

**Female** (Fig. 16A, B, E). Total length 8.57. Carapace 3.60 long, 2.91 wide. Abdomen 4.69 long, 3.09 wide. Clypeus 0.20 high. Eye sizes and inter-distances: AME 0.90, ALE 0.59, PLE 0.50, AERW 2.71, PERW 2.57, EFL 1.77. Legs: I 7.58 (2.20, 1.45, 1.88, 1.25, 0.80), II 6.91 (2.20, 1.30, 1.58, 1.13, 0.70), III 8.54 (2.83, 1.40, 1.75, 1.68, 0.88), IV 7.79 (2.38, 1.10, 1.80, 1.73, 0.78). Habitus (Fig. 16E) similar to that of male except without dense setae on the carapace and with an almost T-shaped yellow area on thorax. Epigyne (Fig. 16A, B). Wider than long, with pair of very shallow posterior hoods; copulatory openings slit-shaped, located medially; copulatory ducts very short, curved medially; spermathecae divided into two chambers, the anterior chamber oval, much smaller than the elongate, posterior chamber.

##### Distribution.

Known only from Hainan Island, China.

#### ﻿Genus Qiongattus gen. nov.

Taxon classificationAnimaliaAraneaeSalticidae

1326513D-6CFF-5F48-8419-BDA03180C27B

https://zoobank.org/28E12F44-1AFB-49C6-BCB2-1AC04C042AC4

##### Type species.

*Qiongattusyuanyeae* sp. nov. from Hainan, China designated herein.

##### Etymology.

The specific name is a combination of Pinyin “Qiong”, the short name of Hainan Province, and *attus*, meaning jumper. The gender is masculine.

##### Diagnosis.

The classification position of *Qiongattus* gen. nov. is uncertain. It resembles that of *Chinattus* Logunov, 1999, and *Habrocestoides* Prószyński, 1992 in having a single retromarginal cheliceral tooth, and similar copulatory organs, but it can be easily distinguished by the following: (1) the narrow cymbium, which is ~ 3× longer than wide in ventral view, vs. wider and less than two times longer than wide in *Chinattus* and *Habrocestoides* (Prószyński 1992: figs 16, 22; Logunov 1999: figs 13, 24, 28, 32, 35 40; Metzner 2023); (2) the basal epigynal plate lacking round structure, vs. present in *Chinattus* and *Habrocestoides* (Prószyński 1992: figs 25, 26; Logunov 1999: figs 17, 19, 22, 45; Metzner 2023); (3) the spermathecae have U-shaped heads, vs. absent in *Chinattus* and *Habrocestoides* (Prószyński 1992: figs 26, 27; Logunov 1999: figs 18, 20, 23, 46; Metzner 2023). The genus also somewhat resembles that of *Phintella* Strand, 1906 in the general shape of palp, but it can be easily distinguished by the absence of tegular bump, and the postero-prolaterally extended posterior lobe, vs. tegular bump medio-retrolaterally located on bulb and posterior lobe is downward or postero-retrolaterally extending in *Phintella* (Żabka 1985: figs 403, 408, 420, 422, 426, 430, 435, 447–450; Metzner 2023).

##### Description.

See the description of generotype.

##### Composition.

The genus is monotypic presently.

##### Distribution.

Known only from Hainan Island, China.

#### ﻿ Qiongattus yuanyeae sp. nov.

Taxon classificationAnimaliaAraneaeSalticidae

D0F107E1-3E0C-5526-8F65-329645A98600

https://zoobank.org/0B87530B-4E63-48D7-80D9-FBCFE1D65DEE

Figs 17 , 18

##### Type material.

***Holotype*** ♂ (IZCAS-Ar44529), China: Hainan: Ledong County, Jianfengling National Nature Reserve, Tianchi (18°44.45′N, 108°57.49′E, ca. 860 m), 18.vii.2007, S.Q. Li leg. ***Paratypes*** 1♂1♀ (IZCAS-Ar44530–44531), same data as holotype; 1♂1♀ (IZCAS-Ar44532–44533), Jianfengling National Nature Reserve (18°44.38′N, 108°51.06′E, ca. 890 m), 19.vii.2007, C.X. Wang leg.; 1♂1♀ (IZCAS-Ar44534–44535), Lingshui County, Diaoluoshan National Nature Reserve, Direction of the Mysterious Tree (18°43.50′N, 108°52.10′E, ca. 920 m), 18.iv.2011, Y.Y. Zhou leg.; 1♀ (IZCAS-Ar44536), Diaoluoshan Resort (18°43.51′N, 108°52.10′E, ca. 920 m), 20.iv.2011, Y.Y. Zhou leg.

##### Etymology.

The specific name is after one of the collectors of the type specimens, Ms. Yuanye Zhou; noun (name) in genitive case.

##### Diagnosis.

**Figure 17.**
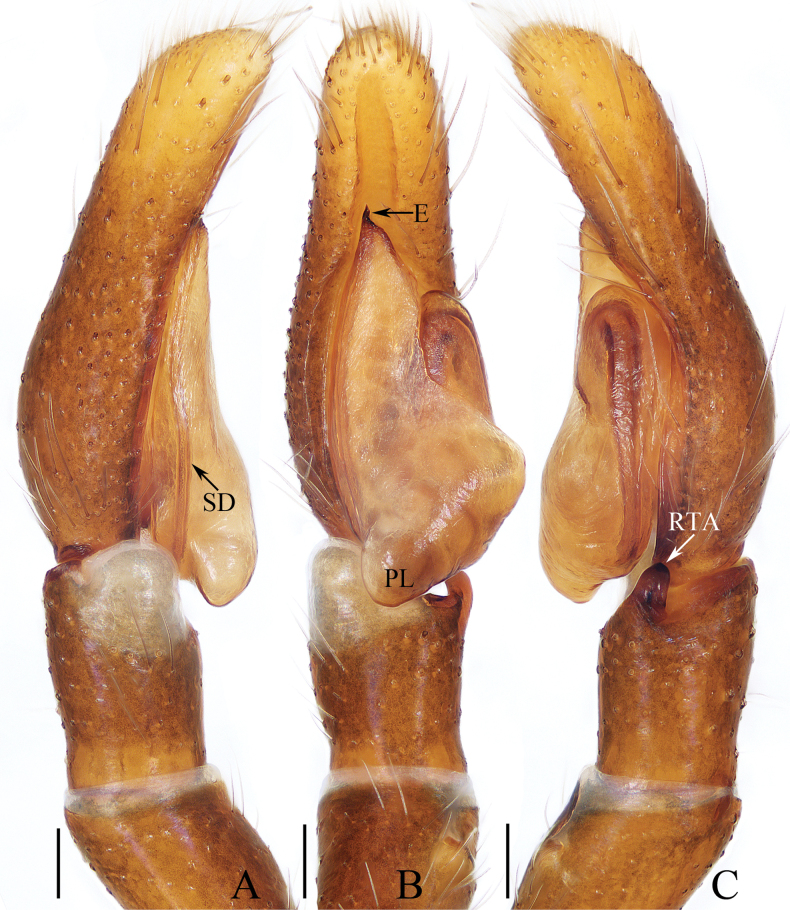
Male palp of *Qiongattusyuanyeae* sp. nov., holotype **A** prolateral **B** ventral **C** retrolateral. Scale bars: 0.1 mm.

**Figure 18.**
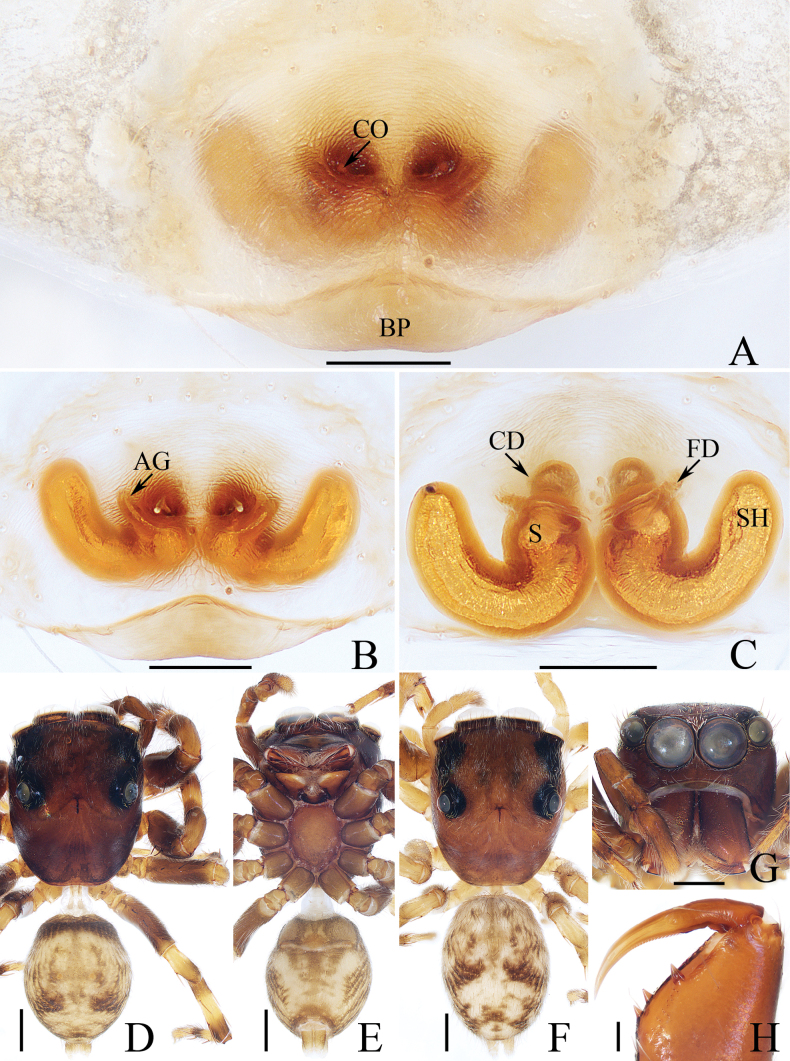
*Qiongattusyuanyeae* sp. nov., male holotype and female paratype **A, B** epigyne, ventral **C** vulva, dorsal **D** holotype habitus, dorsal **E** ditto, ventral **F** female paratype habitus, dorsal **G** holotype carapace, frontal **H** holotype chelicera, posterior. Scale bars: 0.1 mm (**A–C, H**); 0.5 mm (**D–G**).

The male of *Qiongattusyuanyeae* sp. nov. resembles that of *Chinattuschichila* Logunov, 2003 in the general shape of palp, but it can be easily distinguished by the narrow cymbium, which is ~ 3× longer than wide in ventral view (Fig. 17B), vs. less than 2× longer than wide in *C.chichila* (Logunov 2003: fig. 1), and by the RTA, which is acutely narrowed distally in retrolateral view (Fig. 17C), vs. tapered in *C.chichila* (Logunov 2003: fig. 2). The female of this species resembles that of *Chinattusinflatus* Wang & Li, 2020 in having similar basal epigynal plate, but it can be easily distinguished by the presence of spermathecal head and the absence of round structure on basal epigynal plate (Fig. 18A–C) vs. spermathecae lack heads and basal epigynal plate has round structure in *C.inflatus* (Wang and Li 2021: fig. 1A, B).

##### Description.

**Male** (Figs 17, 18D, E, G, H). Total length 3.94. Carapace 2.00 long, 1.65 wide. Abdomen 1.62 long, 1.32 wide. Clypeus 0.12 high. Eye sizes and inter-distances: AME 0.53, ALE 0.31, PLE 0.29, AERW 1.59, PERW 1.50, EFL 1.03. Legs: I 4.38 (1.23, 0.75, 1.00, 0.95, 0.45), II 3.69 (1.13, 0.65, 0.85, 0.63, 0.43), III 4.04 (1.23, 0.63, 0.88, 0.80, 0.50), IV 4.25 (1.32, 0.60, 0.88, 0.95, 0.50). Carapace red-brown to dark brown, covered with dense pale thin setae, with elevated cephalic region, and sloped thorax; fovea longitudinal, dark, bar-shaped. Chelicerae red-brown, each with two promarginal teeth and one retromarginal tooth. Endites widened distally, the distal half of inner margins paler, bearing dense brown setae. Labium red-brown to dark brown. Sternum slightly longer than wide, with straight anterior margin. Legs yellow to dark brown. Abdomen oval, dorsum grey to dark brown, with transverse, antero-marginal dark stripe, pair of anteromedian muscle depressions followed by pair of mediolateral dark patches; venter coloured as dorsum. Palp (Figs 17A–C, 18E). Patella longer than wide, with disto-retrolateral bump; tibia ~ 1.5× longer than wide, with short, inwards curved RTA acutely narrowed to rather pointed tip in retrolateral view; cymbium narrow, ~ 3× longer than wide in ventral view; bulb elongated, swollen medio-posteriorly, with blunt posterior lobe extending postero-prolaterally; embolus originating the apex of bulb, short, slightly curved medially.

**Female** (Fig. 18A–C, F). Total length 4.16. Carapace 2.10 long, 1.81 wide. Abdomen 1.94 long, 1.45 wide. Clypeus 0.12 high. Eye sizes and inter-distances: AME 0.56, ALE 0.32, PLE 0.29, AERW 1.74, PERW 1.64, EFL 1.16. Legs: I 4.44 (1.38, 0.83, 1.05, 0.73, 0.45), II 4.00 (1.25, 0.75, 0.90, 0.65, 0.45), III 4.37 (1.38, 0.65, 0.98, 0.88, 0.48), IV 4.62 (1.43, 0.63, 1.00, 1.03, 0.53). Habitus (Fig. 18F) similar to that of male, except paler. Epigyne (Fig. 18A–C). Wider than long, with broad, arc-shaped basal plate; copulatory openings small, medially located, separated from each other ~ 1/3 the basal plate width; copulatory ducts very short, with bar-shaped accessory glands; spermathecae oval, with almost U-shaped heads.

##### Distribution.

Known only from the type locality in Hainan Island, China.

#### ﻿Genus Spiralembolus gen. nov.

Taxon classificationAnimaliaAraneaeSalticidae

FC8C2F10-DFC6-5D22-9F0C-D0CAE7E198BF

https://zoobank.org/BEE0809E-5C20-4339-9DB5-194F9C9FC275

##### Type species.

*Spiralembolusyinggeling* sp. nov. from Hainan, China designated herein.

##### Etymology.

The specific name is a combination of spiral and embolus, referring to the species that has spiral embolus. The gender is masculine.

##### Diagnosis.

*Spiralembolus* gen. nov. can be recognized as a member of the tribe Euophryini Simon, 1901 for the same reasons as *Logunattus* gen. nov. It is remarkable for the thick, spiral embolus. It above diagnosed with *Logunattus* gen. nov. It also resembles that of *Euochin* Prószyński, 2018 in having similar habitus and palpal structure, especially the presence of white setae on the dorsum of palpal tibia and cymbium, and the oval or round spermathecae, but it can be easily distinguished by the following: (1) the thick, spiralled embolus, not forming a disc at base, vs. flagelliform, coiled embolus, and mostly forming a disc at base in *Euochin* (Zha et al. 2014: figs 5, 8, 16, 19; Metzner 2023); (2) the presence of a cluster of white setae on clypeus, and dorsal abdominal scutum in males, vs. absent in *Euochin* (Zha et al. 2014: figs 2, 13; Metzner 2023); (3) the epigyne lacking pair of oval or round transparent atria, and the concomitant lateral ridges, vs. present in *Euochin* (Zha et al. 2014: figs 3, 10, 14, 21; Metzner 2023). The genus also somewhat resembles that of *Chalcovietnamicus* Marusik, 1991 in having similar epigyne, but it can be easily distinguished by the presence of clusters of white setae on clypeus, and the dorsum of tibia and cymbium of palp, the spiralled embolus lacks lamellar basal apophysis, vs. without clusters of white setae on clypeus, and the dorsum of tibia and cymbium of palp, non-spiralled embolus with lamellar basal apophysis in *Chalcovietnamicus* (Żabka 1985: figs 71–74; Wang and Li 2022a: figs 3A–C, 4F).

##### Description.

Small-sized jumping spider. Sexual dimorphism scarcely evident. Carapace darker in males and, covered with cluster of white setae on clypeus that only presents in males, with elevated, sub-square cephalic region bearing thin setae, and sloped thorax with dark brown stripes; fovea dark red, longitudinal. Chelicerae each with two promarginal teeth and one retromarginal tooth. Endites widened distally, with paler inner margins bearing dense brown setae. Labium tapered, almost linguiform. Sternum longer than wide, with straight anterior margin, bearing brown and pale setae of various lengths. Legs yellow to dark brown, spinous, with clusters of white setae on femora and tibiae that only present in males. Abdomen darker in males, dorsum covered with dense white setae and with antero-median scutum, those only present in males, and with several transverse, pale stripes posteriorly in both sexes; venter setose.

Palp. Tibia very short, covered with dorsal white setae; RTA short, curved medially or distally, with rather blunt tip; cymbium longer than wide, covered with dorsal white setae on proximal half; bulb swollen; embolus thick, strongly sclerotized, originates from the prolatero-apical portion of bulb, spiralled into coils.

Epigyne. Wider than long, without distinct atrium; copulatory openings located anteriorly or medially, round or oval, separated from each other at least their width; copulatory ducts short, strongly curved anteriorly; spermathecae oval or spherical, with distinct Bennett’s glands; fertilization ducts originate from the anterior portions of spermathecae, extending transversely.

##### Composition.

The genus only contains two species, the generotype, and *S.yui* sp. nov.

##### Distribution.

Known only from Hainan Island, China.

#### ﻿ Spiralembolus yinggeling sp. nov.

Taxon classificationAnimaliaAraneaeSalticidae

2B92D2F9-3086-5878-BD5B-99F0FB8A22A0

https://zoobank.org/FE06A9FC-91BA-453A-9DF5-85722794EA3D

Figs 19 , 20

##### Type material.

***Holotype*** ♂ (IZCAS-Ar44537), China: Hainan: Qiongzhong County, Yinggeling National Nature Reserve, Yinggezui Waterfall (19°03.04′N, 109°44.89′E, ca. 620 m), 8.v.2011, Y.Y. Zhou leg. ***Paratypes*** 1♂3♀ (IZCAS-Ar44538–44541), same data as holotype; 3♀ (IZCAS-Ar44542–44544), Changjiang County, Bawangling National Nature Reserve, Dong’er Management Station (19°05.18′N, 109°11.80′E, ca. 800 m), 25.xii.2007, C.X. Wang leg.; 11♂10♀ (IZCAS-Ar44545–44565), Qiongzhong County, Limushan National Nature Reserve, Direction of Shuiba (19°10.87′N, 109°45.32′E, ca. 960 m), 5.v.2011, Y.Y. Zhou leg.; 1♂2♀ (IZCAS-Ar44566–44568), Wuzhishan City, Wuzhishan National Nature Reserve, Direction of Yunding (18°54.42′N, 109°40.65′E, ca. 1140 m), 27.iv.2011, Y.Y. Zhou leg.

##### Etymology.

The specific name is after the holotype locality, Yinggeling National Nature Reserve ; noun (name) in apposition.

##### Diagnosis.

**Figure 19.**
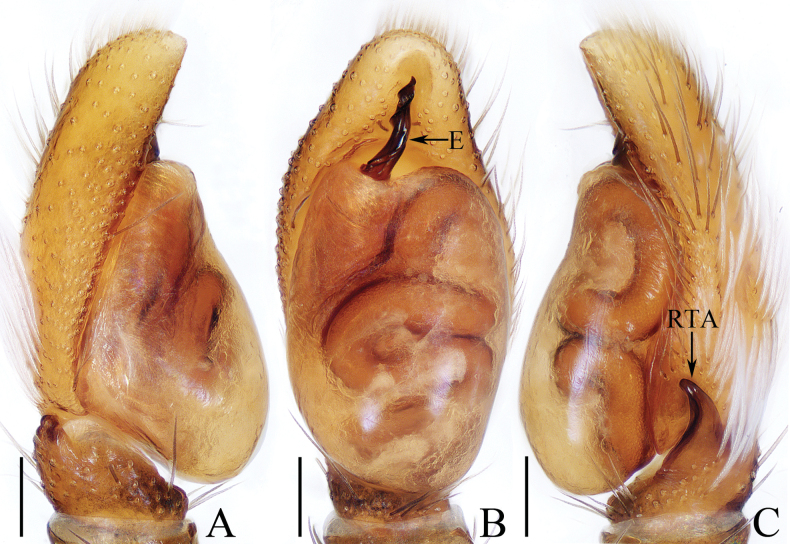
Male palp of *Spiralembolusyinggeling* sp. nov., holotype **A** prolateral **B** ventral **C** retrolateral. Scale bars: 0.1 mm.

**Figure 20.**
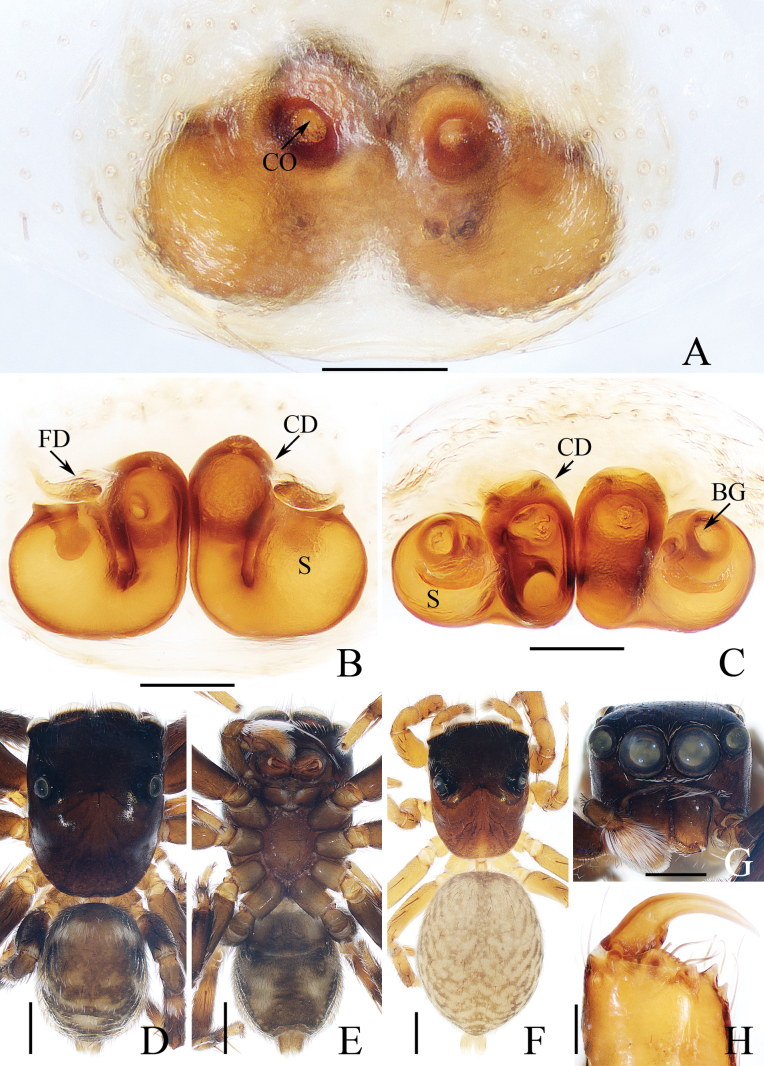
*Spiralembolusyinggeling* sp. nov., male holotype and female paratype **A** epigyne, ventral **B** vulva, dorsal **C** vulva, anterodorsal **D** holotype habitus, dorsal **E** ditto, ventral **F** female paratype habitus, dorsal **G** holotype carapace, frontal **H** holotype chelicera, posterior. Scale bars: 0.1 mm (**A–C, H**); 0.5 mm (**D–G**).

**Figure 21.**
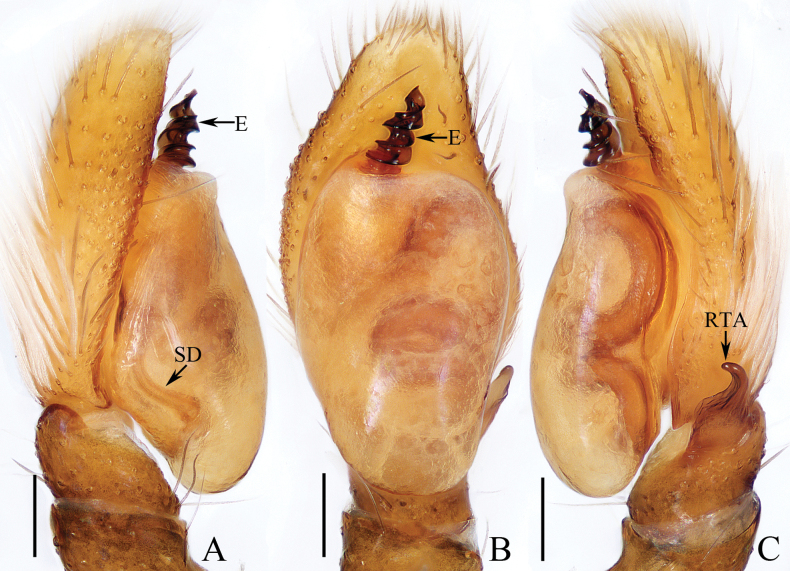
Male palp of *Spiralembolusyui* sp. nov., holotype **A** prolateral **B** ventral **C** retrolateral. Scale bars: 0.1 mm.

**Figure 22.**
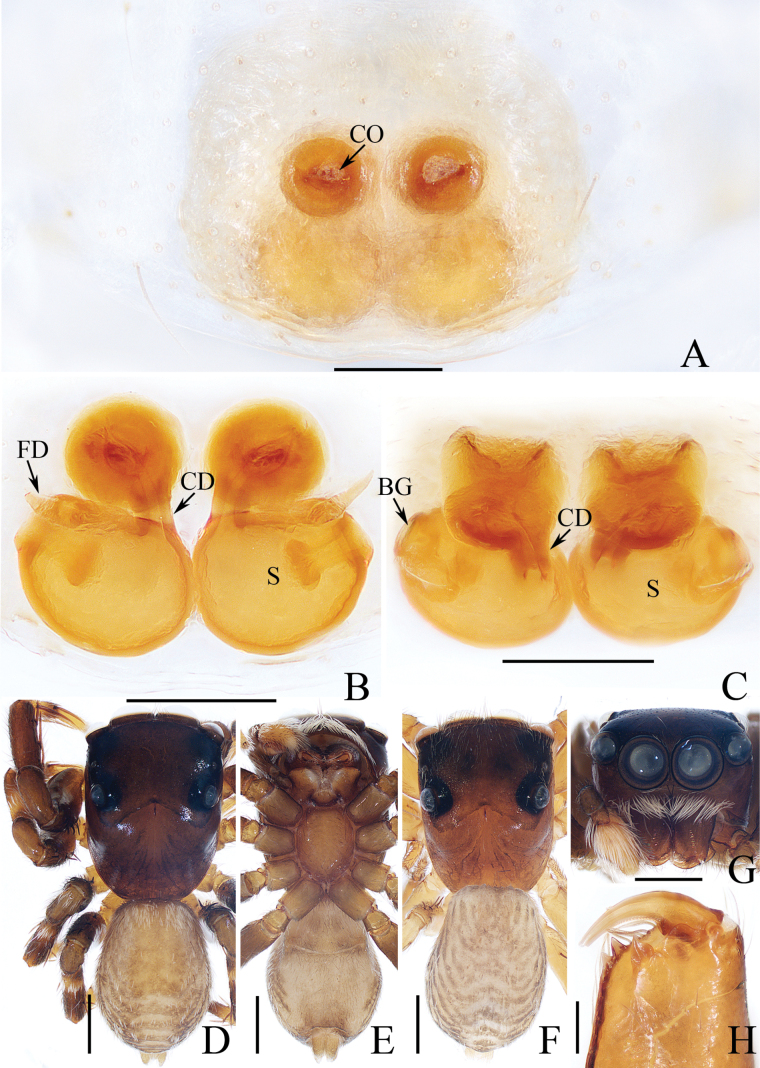
*Spiralembolusyui* sp. nov., male holotype and female paratype **A** epigyne, ventral **B** vulva, dorsal **C** vulva, anterodorsal **D** holotype habitus, dorsal **E** ditto, ventral **F** female paratype habitus, dorsal **G** holotype carapace, frontal **H** holotype chelicera, posterior. Scale bars: 0.1 mm (**A–C, H**); 0.5 mm (**D–G**).

*Spiralembolusyinggeling* sp. nov. resembles that of *S.yui* sp. nov. in having similar habitus and copulatory organs, but it can be easily distinguished by the following: (1) the embolus is spiralled into less than three coils (Fig. 19B), vs. more than four coils in *S.yui* (Fig. 21B); (2) the RTA is tapered in retrolateral view (Fig. 19C), vs. acutely narrowed postero-medially in *S.yui* (Fig. 21C); (3) the copulatory ducts ca. half the spermathecal width, and connected to the postero-inner portions of spermathecae (Fig. 20B), vs. ~ 1/4 the spermathecal width, and connected to the antero-inner portions of spermathecae in *S.yui* (Fig. 22B).

##### Description.

**Male** (Figs 19, 20D, E, G, H). Total length 3.20. Carapace 1.76 long, 1.36 wide. Abdomen 1.40 long, 1.10 wide. Clypeus 0.07 high. Eye sizes and inter-distances: AME 0.40, ALE 0.26, PLE 0.22, AERW 1.36, PERW 1.28, EFL 0.80. Legs: I 3.26 (1.00, 0.55, 0.80, 0.53, 0.38), II 2.82 (0.90, 0.48, 0.63, 0.43, 0.38), III 3.24 (1.03, 0.48, 0.75, 0.63, 0.35), IV 3.57 (1.08, 0.48, 0.80, 0.83, 0.38). Carapace red-brown to dark, almost square, covered with thin setae on cephalic region, with cluster of white setae on clypeus, and oblique, dark brown stripes on thorax; fovea longitudinal, dark, linear. Chelicerae dark yellow to red-brown, each with two promarginal teeth and one retromarginal tooth. Endites almost square, with paler inner margins bearing brown setae. Labium tapered, bearing several dark setae on paler distal portion. Sternum almost oval, yellow-brown, and covered with dark setae. Legs yellow to dark brown, bearing dense dark brown ventral setae on tibiae I and clusters of white setae on femora and tibiae. Abdomen oval, dorsum dark brown, covered with white and dark setae and anteromedian scutum, with irregular pale-yellow patches medially and transverse, pale stripes posteriorly; venter dark brown, covered with short dark brown setae, with pair of mediolateral pale patches. Palp (Figs 19A–C, 20E). Tibia short, covered with dense white, long setae dorsally, with tapered RTA curved towards ventral side medially and blunt apically; cymbium ~ two times longer than wide, covered with dense, long, white setae on the dorsum of proximal half; bulb swollen; sperm duct strongly curved retrolaterally; embolus originates from the antero-prolateral portion of bulb, spiralled into less than three coils, with rather pointed tip.

**Female** (Fig. 20A–C, F). Total length 3.68. Carapace 1.56 long, 1.20 wide. Abdomen 1.98 long, 1.54 wide. Clypeus 0.08 high. Eye sizes and inter-distances: AME 0.39, ALE 0.24, PLE 0.22, AERW 1.24, PERW 1.17, EFL 0.78. Legs: I 3.13 (0.95, 0.55, 0.75, 0.50, 0.38), II 2.90 (0.93, 0.48, 0.63, 0.48, 0.38), III 3.36 (1.05, 0.50, 0.78, 0.60, 0.43), IV 3.79 (1.08, 0.50, 0.88, 0.80, 0.53). Habitus (Fig. 20F) similar to that of male except paler, without dense dorsal white and dark setae, and the dorsal scutum on the abdomen, the cluster of white setae on clypeus, and clusters of white setae on femora and tibiae of legs. Epigyne (Fig. 20A–C). Wider than long; copulatory openings round, anteromedially located, separated from each other ~ 1.5× equal to their width; copulatory ducts thick, inner to spermathecae, close to each other, strongly curved anteromedially, and connected to the posterior portions of spermathecae; spermathecae almost oval, ~ 2× wider than copulatory ducts; fertilization ducts originate from the most antero-inner portions of spermathecae, transversely extending.

##### Distribution.

Known only from the type locality in Hainan Island, China.

#### ﻿ Spiralembolus yui sp. nov.

Taxon classificationAnimaliaAraneaeSalticidae

A996FD99-3E5C-58C6-ADAF-F898C50A1624

https://zoobank.org/2FEF8AC3-C46B-4A0F-88F1-F318CAD52128

Figs 21 , 22

##### Type material.

***Holotype*** ♂ (IZCAS-Ar44569), China: Hainan: Ledong County, Jianfengling National Nature Reserve, Wufenqu (18°44.03′N, 108°55.46′E, ca. 960 m), 15.viii.2010, G. Zheng leg. ***Paratypes*** 1♂2♀ (IZCAS-Ar44570–44572), same data as holotype; 4♂2♀ (IZCAS-Ar44573–44578), Wuzhishan City, Wuzhishan National Nature Reserve, Direction of Yunding (18°54.42′N, 109°40.65′E, ca. 1140 m), 27.iv.2011, Y.Y. Zhou leg.

##### Etymology.

The specific name is after Mr. Yu Li (937–978), one of the famous ancient Chinese poets and one sorrowful ancient Chinese Emperor; noun (name) in genitive case.

##### Diagnosis.

*Spiralembolusyui* sp. nov. closely resembles that of *S.yinggeling* sp. nov., but it can be distinguished by the following: (1) the embolus is spiralled into more than four coils (Fig. 21B), vs. less than three coils in *S.yinggeling* (Fig. 19B); (2) the RTA is acutely narrowed postero-medially in retrolateral view (Fig. 21C), vs. tapered in *S.yinggeling* (Fig. 19C); (3) the copulatory ducts are ~ 1/4 the spermathecal width and connected to the antero-inner portions of spermathecae (Fig. 22B), vs. ~ half the spermathecal width, and connected to the postero-inner portions of spermathecae in *S.yinggeling* (Fig. 20B).

##### Description.

**Male** (Figs 21, 22D, E, G, H). Total length 2.83. Carapace 1.56 long, 1.17 wide. Abdomen 1.31 long, 0.98 wide. Clypeus 0.09 high. Eye sizes and inter-distances: AME 0.39, ALE 0.23, PLE 0.20, AERW 1.19, PERW 1.10, EFL 0.75. Legs: I 3.13 (0.95, 0.55, 0.78. 0.45, 0.40), II 2.66 (0.83, 0.43, 0.60, 0.45, 0.35), III 3.11 (1.00, 0.45, 0.68, 0.60, 0.38), IV 3.39 (1.08, 0.50, 0.73, 0.70, 0.38). Carapace red brown except the lateral of the eye field black, covered with dense white setae on clypeus and with oblique, dark stripes on the sloped thorax; fovea longitudinal, dark, linear. Chelicerae yellow to yellow-brown, each with two promarginal teeth and one retromarginal tooth. Endites widened medio-posteriorly, bearing brown setae on the pale distal half of inner margins. Labium tapered, with several dark brown setae at distal portion. Sternum yellow-brown, ~ 1.5× longer than wide, with straight anterior margin, covered with sparse dark setae of various lengths. Legs yellow to dark brown, with clusters of white setae on femora and tibiae. Abdomen oval, dorsum covered with dense white, short setae and sparse brown setae, with anteromedian scutum ~ half the abdomen width and length, and four transverse pale stripes posteriorly; venter pale to brown, covered with brown setae. Palp (Figs 21A–C, 22E). Tibia wider than long; RTA lamellar baso-medially, the remainder acutely narrowed and curved ventrally, and with rather pointed tip; cymbium longer than wide, covered with dense white setae on dorsum of the proximal half; bulb elongate-oval, swollen; sperm duct strongly curved retrolaterally; embolus originates from the antero-prolateral portion of bulb, spiralled into more than four coils, with pointed tip.

**Female** (Fig. 22A–C, F). Total length 2.85. Carapace 1.57 long, 1.15 wide. Abdomen 1.45 long, 1.06 wide. Clypeus 0.09 high. Eye sizes and inter-distances: AME 0.38, ALE 0.23, PLE 0.21, AERW 1.21, PERW 1.13, EFL 0.74. Legs: I 2.66 (0.80, 0.48, 0.63, 0.40, 0.35), II 2.35 (0.75, 0.40, 0.55, 0.35, 0.30), III 2.74 (0.83, 0.43, 0.60, 0.53, 0.35), IV 3.14 (0.93, 0.40, 0.73, 0.70, 0.38). Habitus (Fig. 22F) similar to that of male except paler, without the dense white setae on clypeus and the dorsal scutum on abdomen. Epigyne (Fig. 22A–C). Almost as long as wide; copulatory openings oval, medially located, separated from each other ca. their width; copulatory ducts short, connected to the antero-inner portions of spermathecae; spermathecae oval, touched; fertilization ducts lamellar, transverse extending.

##### Distribution.

Known only from the type locality in Hainan Island, China.

## Supplementary Material

XML Treatment for Carrhotus qingzhaoae

XML Treatment for Gedea liangweii

XML Treatment for Heliophanoides moi

XML Treatment for Indopadilla songi

XML Treatment for Logunattus

XML Treatment for Logunattus dufui

XML Treatment for Logunattus libaii

XML Treatment for Myrmarachne mixiaoqii

XML Treatment for Nandicius shihaitaoi

XML Treatment for Pancorius hainanensis

XML Treatment for Qiongattus

XML Treatment for Qiongattus yuanyeae

XML Treatment for Spiralembolus

XML Treatment for Spiralembolus yinggeling

XML Treatment for Spiralembolus yui

## ﻿Additional information

### Author ORCIDs

Shuqiang Li https://orcid.org/0000-0002-3290-5416

### Data availability

All of the data that support the findings of this study are available in the main text or Supplementary Information.
